# The 95% CDIRAs, a Credible Interval Based Method to Capture Uncertainty in Population Modeling: Pharmacokinetics Versus Sum of Exponentials Case Study

**DOI:** 10.1002/psp4.70326

**Published:** 2026-08-27

**Authors:** Linda Wanika, Ine Skottheim Rusten, James Kermode, Michael J. Chappell

**Affiliations:** ^1^ School of Engineering University of Warwick Coventry UK; ^2^ Systems Resource Lab AS Kragerø Norway

**Keywords:** Bayesian, mixed effects modeling, pharmacokinetics, uncertainty quantification

## Abstract

Determining the credibility of population PK/PD models is a challenge, particularly when performing uncertainty quantification (UQ) for parameter estimates, which is often represented through relative standard error (RSE) values. It is important to note that RSE values are primarily based on the variance of the parameter distribution and is therefore less sensitive to the overall shape and quantiles of a distribution which also reflects the uncertainty of a parameter. This can lead to unreliable uncertainty results and misleading interpretations. Robust UQ is important for model parameter estimation, as the results from in silico modeling are often used to inform subsequent steps in drug development. To capture the overall parameter uncertainty obtained from parametric approaches, the 95% credible interval ratios (95% CDIRAs) are introduced. The 95% CDIRAs only require the distribution for the individual parameter and consider the shape and quantiles of the distribution in addition to the variance. To showcase the 95% CDIRAs, an exemplar case study comprising simulated plasma concentration data was analyzed to assess whether a one compartment absorption population PK model or a reparameterized sum of exponentials (SOE) model can provide a more credible fit to plasma concentration data. Both metrics identified the population PK model as a more credible fit compared to the reparameterized SOE model. However, on occasions the precision classifications of the RSE values were higher than the adopted 95% CDIRAs precision classifications, which further indicates that uncertainty assessment based on variance alone does not necessarily encompass the full level of uncertainty for a parameter estimate.

## Study Highlights

- What Is the Current Knowledge on the Topic?
  ○ Uncertainty quantification (UQ) remains a challenge in PK/PD modeling. While relative standard errors (RSEs) are often used to approximate uncertainty, RSEs primarily focus on the variance of the distribution and not the overall shape, which can lead to unreliable precision results.
- What Question Did This Study Address?
  ○ The aim of this study was to introduce and showcase the credible interval‐based UQ measure, the 95% credible interval ratios (95% CDIRAs) in comparison to the relative standard errors as an additional measure of uncertainty and credibility. The case study exemplar question was, “Which population model would provide a more credible and robust fit to plasma concentrations, a PK model or a sum of exponentials model?.”
- What Does This Study Add to Our Knowledge?
  ○ The 95% CDIRAs highlights parameter uncertainties. Moreover, the 95% CDIRAs precision results showcase the over‐precision predictability of RSE classifications. For the PK and SOE model parameterisation examples, this study showcases that the same algebraic model structure can still yield different uncertainties which may be amplified through Jensen's inequality.
- How Might This Change Drug Discovery, Development, and/or Therapeutics?
  ○ The 95% CDIRAs measure provides a credible approach to uncertainty assessment for parametric PKPD modeling and can provide more credible precision results for parameter estimations. Additionally, the lower and upper bounds of the 95% CDIRAs can be used to highlight which sides of an estimated distribution have more uncertainty, which can aid in iterative model development and assessment.

## 1 Introduction

Population modeling is widely used to estimate parameter values in pharmacokinetic/pharmacodynamic (PK/PD) models and has advanced many drug developments [1, 2, 3]. Despite this success, credibility assessment of model development and uncertainty quantification (UQ) analysis of these models remains challenging [4, 5, 6, 7, 8, 9, 10, 11, 12, 13].

Uncertainty can be described as the unknown knowledge in a process. There are two main types of uncertainty: aleatoric (data noise) and epistemic (model system) uncertainty [14]. For population modeling, uncertainty arises in all the steps from data selection to model prediction. Model parameter estimates are directly linked to the model development, validation, and subsequent modeling analyses.

In PKPD population modeling, aleatoric uncertainty is often represented as residual unexplained variability (RUV) and between subject variability (BSV) is attributed to epistemic uncertainty, but this metric simply yields variability [2, 15]. Relative standard error (RSE) values are an UQ metric for parameter estimations [16, 17]. Low RSE values indicates narrow distributions centred to the parameter estimates. High RSE values indicates wide distributions which encompass more uncertainty as many values for that parameter could be used to yield the same model fits.

Some RSE approximation methods such as the stochastic approximation, linearisation and first order conditional estimation methods rely on second derivative calculations which may fail [18, 19, 20, 21]. RSE values only consider the distribution variance and not the distribution's shape or quantiles, which describe the overall uncertainty. These restrictions can lead to misrepresentations of the overall uncertainty of a parameter.

This paper introduces 95% credible interval ratios (95% CDIRAs) which is not entirely dependent on the variance of a distribution or the estimation of second derivatives. Additionally, the 95% CDIRA values for positively constrained parameters are bounded between 0 and 1. To showcase this new UQ metric, this paper will investigate whether a population PK or a reparameterized sum of exponentials (SOE) version of this model could be considered as a more credible model for the representation of simulated plasma concentrations using both RSEs and 95% CDIRAs.

## 2 Background

### 2.1 Question Centric Approach for Parameter Estimation Uncertainty Quantification

To identify a suitable metric a question centric approach was used which focuses on the rationale and evidence for the underlying question of interest(s) (QOIs) [22]. In order to determine which population model is more credible, the main QOI is: “What is the uncertainty associated with the model parameters for both the population PK and reparameterized SOE models?.”

Any UQ metric which may be used to assess the credibility of parameter estimation should be able to answer the following questions:

1. What method was used to derive this uncertainty?
2. Which aspects of a parameter distribution is attributed in this metric?
3. How is this metric related to the credibility of the parameter estimates?

**FIGURE 1:**
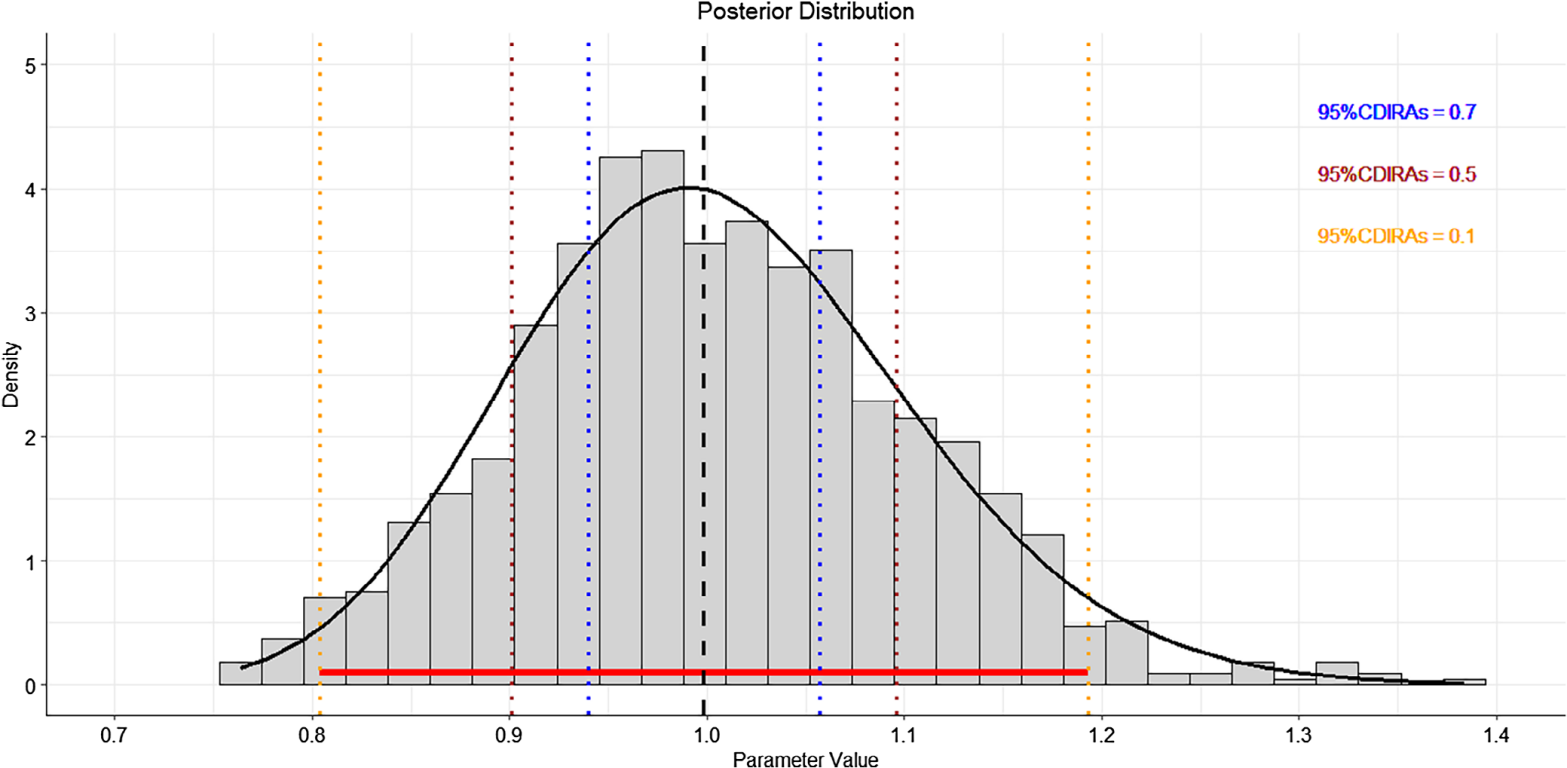
Typical parameter distribution. A typical histogram of the posterior distribution for a parameter. Most of the draws for this example parameter estimate fall between 0.925 and 1.050, which is within the boundary for 0.7 for high precision based on the 95% credible interval ratio averages. As the 95% credible interval ratio averages become wider, more of the distribution is encompassed; however, these estimates are further deviated from the median and the credible interval.

Typically, parametric parameter estimation is often derived from a postulated posterior distribution. Figure 1 shows a typical posterior distribution which encompasses the uncertainty of a parameter estimate:

The distribution displays the possible values for a particular parameter and the frequency at which a particular parameter interval was chosen to sample from, to estimate the likelihood of the model. In Figure 1, the 95% credible interval (CDI (red line)) encompasses the parameter estimate (the median (dashed line)) as well as the estimates which account for 95% of the area under the distribution.

The 95% CDI is an established uncertainty quantification metric [23]. There are two main intervals that can be used to calculate the 95% CDI, the highest density interval (HDI) or the equal‐tailed interval (EDI) [24]. The HDI was chosen here as this interval considers the entire distribution and thus provides a more accurate representation of the 95% CDI, whereas the EDI eliminates 2.5% of the distribution from each tail end. A short 95% CDI indicates a narrow distribution, similar to a low RSE value, and can be interpreted as offering less uncertainty. In contrast, a wide 95% CDI reduces the model and parameter estimate credibility due to increased uncertainty [25].

The 95% CDI is a routinely used uncertainty metric and already expresses the quantiles that were used to derive a parameter estimate. This metric can be combined with the estimate to identify posterior distribution regions which are more uncertain than others.

Figure 1 showcases the 95% CDI ratio averages (95% CDIRAs) that are derived from the 95% CDI and which can also be used to identify regions in parameter space that are more uncertain than others. The higher the 95% CDIRAs, the narrower the spread between the parameter estimate and the 95% CDI bounds.

### 2.2 Relative Standard Errors

In typical PK/PD modeling, RSEs are typically defined as the standard error of a parameter estimate divided by the parameter estimate (Equations 11 and 12). The standard error can be estimated using approximation methods for the Fisher's Information matrix (FIM), which considers the variance of the parameter estimates in a given parameter vector [18, 19, 20, 21]. The standard error can also be estimated using the covariance matrix, which has an inverse relationship with the FIM [20]. The spread of the values within the distribution with respect to the estimate can be assessed for precision. RSE values can be classified into low, moderate, or high precision [26, 27, 28].

While the classification and boundary tend to vary across studies, for this study, the precision classification for the RSE will be based as follows: ≤ 30% = high precision, 30%–50% = moderate precision, 50%–100% = low precision, ≥ 100% = very low precision.

## 3 Methods

### 3.1 Data

**FIGURE 2:**
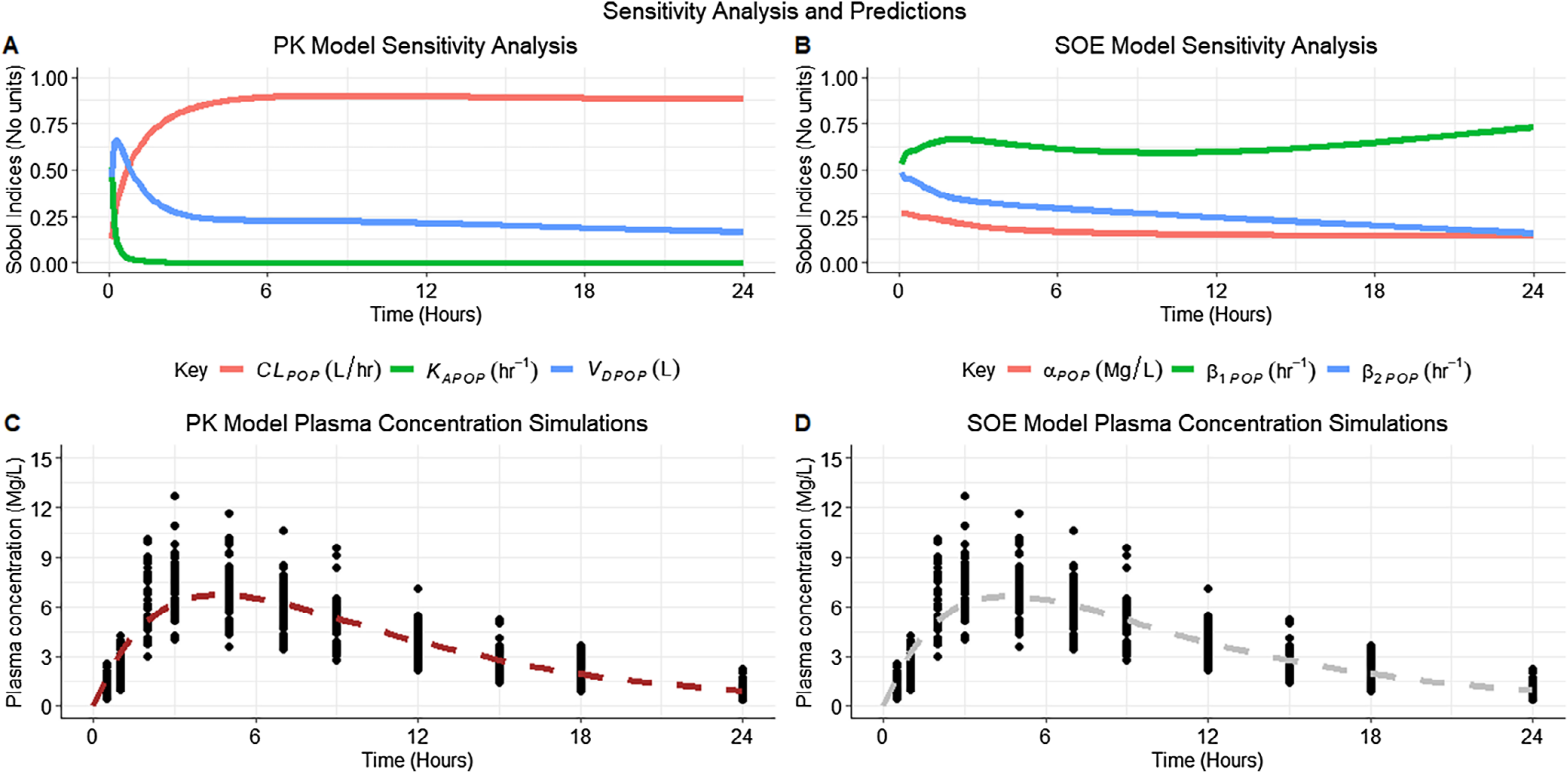
Sensitivity analyses and model predictions for PK and SOE models. Sobol indices for both the PK (A) and SOE (B) models for the simulated plasma concentration. The plots visualize how sensitive the model predicted plasma concentration outputs are to the model parameters over a 24‐h period based on an initial dose of 100 mg. The model predictions for the PK (C) and SOE (D) models compare the model predictions based on the median parameter estimates compared to the individual observed simulated plasma concentrations over a 24‐h period.

The Monolix demo data from the Oral1_project was used in this study. This dataset can be accessed in Monolix as follows: Monolix2024R1/demos/6.PK_models/6.1.single_route/data/oral_data.txt. Monolix is free to download and install for academic use [29]. Briefly, 50 adult patients were administered a single simulated dose of 100 mg, and the simulated plasma concentration levels were then sampled 11 times (0.5, 1, 2, 3, 5, 7, 9, 12, 15, 18, and 24 h (Figure 2)).

### 3.2 Epistemic Uncertainty Analysis

There are several steps that should be considered prior to model fitting to ensure that the model utilized is appropriate and sufficiently robust, such as sensitivity analysis and structural identifiability analysis [30, 31, 32, 33]. Global sensitivity analysis assesses the impact of perturbations to model parameters (across the entire parameter space) on the model's outputs [34]. Structural identifiability analysis (SIA) is used to determine whether a unique, finite or countable set of parameter values can be used to yield the same model output(s). Thus, if a parameter is classed as structurally unidentifiable then this increases the level of uncertainty and reduces the reliability of parameter estimates and for that model as effectively any value for such a parameter can yield the same model output(s). In this paper, global sensitivity analysis was performed using Sobol Indices and SIA was performed using the Taylor Series approach [30]. It can be readily shown that both models are structurally identifiable (see Supporting Information S1).

### 3.3 Structural Models Used in the Study

A one compartment absorption PK ordinary differential equation model and a reparameterized sum of exponentials (SOE) version of this model were used to describe the simulated plasma concentration data. This simple PK model (Monolix Oral1 demo project's default model) was chosen to demonstrate that even the algebraically equivalent SOE model can produce different sensitivity and uncertainty analyses due to changes in the parameter combinations. More complex PK models (e.g., time‐delay or transit compartment models) would complicate the identifiability analysis and require additional modifications to the corresponding SOE model.

The system of ordinary differential equations (ODEs) which describe the PK model is described below:

(1) $$\frac{d\left(\text{Depot}\right)}{dt}=-K_{A}\cdot \text{Depot}\left(t\right)$$

(2) $$\frac{d\left(\text{Central}\right)}{dt}=K_{A}\cdot \text{Depot}\left(t\right)-\frac{\mathrm{CL}}{V_{D}}\cdot \text{Central}\left(t\right)$$

(3) $$y\left(t\right)=\frac{\text{Central}\left(t\right)}{V_{D}}$$

The initial conditions are as follows: Depot(0) = 100 mg and Central(0) = 0 mg. $y\left(t\right)$, refers to the output of the ODE system which represents the observed plasma concentration. Depot and Central are the state variables which are used to represent the changes to the drug in the GI tract (Depot) and in the plasma (Central). The parameters $K_{A}\;\left(h^{-1}\right)$, CL (L/h), and $V_{D}$ (L) refer to the absorption rate, the clearance and the volume of distribution respectively. Given that the observed profile is the simulated plasma concentration, the model output is the quantity of the simulated drug in the Central compartment divided by $V_{D}$ as given by Equation (3).

The SOE model is defined as follows:

(4) $$y\left(t\right)=\alpha \cdot e^{-\beta_{1}\cdot t}-\alpha \cdot e^{-\beta_{2}\cdot t}$$

where $y\left(t\right)$, represents the plasma concentration simulation, and α (Mg/L), $\beta_{1}$ $\left(h^{-1}\right)$, and $\beta_{2}$ $\left(h^{-1}\right)$, are the parameters of the SOE model. The initial condition for the plasma concentration is 0 mg. Equation (4) is effectively a reparameterized version of the solution of Equation (3):

(5) $$y\left(t\right)=\frac{100}{V_{D}}\cdot \frac{K_{A}}{K_{A}-\frac{\mathrm{CL}}{V_{D}}}\cdot \left(e^{-\frac{\mathrm{CL}}{V_{D}}\cdot t}-e^{-K_{A}\cdot t}\right)$$

The parameters, $\beta_{1}$ and $\beta_{2}$ in the SOE model correspond to $\frac{\mathrm{CL}}{V_{D}}$ and $K_{A}$ respectively. However, the parameter α is a combination of all the parameters including the dose. Although both models are algebraically equivalent, due to Jensen's inequality the parameter estimates for the two models may not yield a 1:1 comparison, as the estimation of individual parameters will be different to the estimation of the parameter combinations [35].

### 3.4 Population Modeling in Stan

To estimate the posterior distribution, the Bayesian programming language Stan was used [36]. To estimate the population parameter posterior distributions, the corresponding prior distribution and the likelihood model need to be defined.

The prior distributions refer to any prior knowledge of the parameters to be estimated prior to the estimation process utilizing the data [37]. The parameters which are estimated include typical population parameter estimates, the ω estimates (BSV), the individual parameter estimates, the error model constants (RUV), and the random effects (η estimates).

A summary of the prior distributions used for both population models can be found in Table 2.

All the parameters were estimated using either a lognormal or a positively constrained normal distribution. Lognormal distributions are typically applied for positively constrained PKPD parameters [38]. Normal distributions are a safe choice for weakly informative priors and are the standard distribution of choice for the ηi parameters [39, 40]. The initial standard deviation values for both population parameters and the ω were set to one, which is often the default value for a normal distribution. However, this resulted in across mode disagreements (multimodal peaks) for the parameters $K_{A_{\mathrm{POP}}}$ and $V_{D_{\mathrm{POP}}}$, due to the flip‐flop kinetics, as well as high.

$\hat{R}$ values and low effective sample size diagnostic values (see diagnostic performance and Table 3). This issue was fixed by decreasing the standard deviation to 0.3 and 0.5 for $K_{A_{\mathrm{POP}}}$ and all other population parameters and ω's respectively, as well as the inclusion of more warmup draw.

In Monolix, the default error model for the Oral1 project is the combined error model which is given by (in ODE form):

(6) $$y\left(t\right)=\frac{\text{Central}\left(t\right)}{V_{D}}+\left(a+b\cdot \frac{\text{Central}\left(t\right)}{V_{D}}\right)\;\cdot \text{Normal}\left(0,1\right)$$

where a and b are the constant and proportional error terms and $\text{Normal}\left(0,1\right)$ refers to a normal distribution with a mean of 0 and standard deviation of 1. The default value for a and b in Monolix are 1 and 0.3 which is reflected in the standard deviation of their respective prior distributions. Lastly, using $K_{A}$ as an example, the individual parameters were expressed as follows:

(7) $$K_{A_{i}}=K_{A_{\mathrm{POP}}}\cdot e^{K_{A_{\mathrm{\eta i}}}}$$

where $K_{A_{i}}$ is the individual parameter estimate for $K_{A}$. $K_{A_{\mathrm{POP}}}$ is the typical population parameter estimate and $K_{A_{\mathrm{\eta i}}}$ refers to the individual random effects associated with $K_{A}$.

The initial values used for all parameters (including the SOE parameters) were set to one, except for the parameter $V_{D}$, which in the Monolix Oral1 project is set to 10.

The likelihood models used for the population PK and SOE models are provided below:

(8) $$y\left(t\right)\sim \text{Normal}\left(\frac{\text{Central}\left(t\right)}{V_{D_{i}}},\left(a+b\cdot \frac{\text{Central}\left(t\right)}{V_{D_{i}}}\right)\right)$$

(9) $$y\left(t\right)\sim \text{Normal}\left(\alpha_{i}\cdot e^{-\beta_{1i}\cdot t}-\alpha_{i}\cdot e^{-\beta_{2i}\cdot t}\right),\left(a+b\cdot \left(\alpha_{i}\cdot e^{-\beta_{1i}\cdot t}-\alpha_{i}\cdot e^{-\beta_{2i}\cdot t}\right)\right)$$

A normal distribution was adopted for the likelihood models as it is the most commonly used distribution of choice for such in population modeling [41].

As the prior distributions and likelihood models are clearly defined, the posterior distributions for the parameters can be estimated using the Hamiltonian Monte Carlo (HMC) algorithm [42]. The estimated posterior distribution is represented as an energy‐based system which can be represented as follows:

(10) $$P\left(H\left(\theta ,m\right)\right)\propto \left(\text{Likelihood distribution}\cdot \text{Prior distribution}\;\right)\cdot e^{-\frac{m^{2}}{2}}$$

where *H* refers to the Hamiltonian, θ refers to the estimated parameter of interest and m is the momentum variable [43]. The exponential term can be represented as m ~ Normal(0,1). Briefly, to begin the HMC process, a sample value form is drawn from the distribution which is then used to solve for θ. This new value will update the proposed values for m and subsequently generate a new value for θ. The two new values for *m* and θ are then either accepted or rejected using an acceptance ratio. If accepted, then the current values are used to propose new values for m and θ, If rejected, values are proposed from the previous values adopted for *m* and θ or from Normal(0,1). This process is repeated until the maximum number of prescribed iterations has been reached, and the trajectory of this process is the estimated (non‐standard form) posterior distribution.

### 3.5 Diagnostic Performance in Stan

In Stan, the diagnostic performances of each chain and each parameter estimation can be assessed. In this study, the measures were r‐hat.

($\hat{R}$), which compares the estimates within each chain to the estimates between each chain, the effective sample size bulk (ESS_Bulk) which estimates the sampling efficiency related to the mean and median of the distributions, and the effective sample size tail (ESS_Tail) which estimates the sampling efficiency related to the tails of the distributions. For good convergence, $\hat{R}$, should be < 1.01 and the ESS measures should be > 100 per Markov chain (> 400 in total) [43].

### 3.6 RSE Estimations in Stan

The RSE estimation used in this study is based on the covariance matrix. This is derived from the variance calculations of the posterior parameter estimates.

(11) $$\mathrm{SE}=\sqrt{\text{Diagonal elements of covariance matrix}}$$

(12) $$\mathrm{RSE}=\frac{\mathrm{SE}}{\text{Median nsparameter estimate}}\times 100$$

The median estimate was chosen as this provides more information regarding the overall shape of the distribution when combined with the 95% CDI.

### 3.7 The 95% CDIRAs

This 95% CDI based metric can be readily derived from the posterior distribution. The proposed equations for the 95% CDI ratio lower bound (95% CDIRLB) and the 95% CDI ratio upper bound (95% CDIRUB) are as follows:

(13) $$95\%\text{CDIRALB}=\frac{95\%\text{CDILB}}{\text{median parameter estimate}}.$$

(14) $$95\%\text{CDIRAUB}=\frac{\;\text{median parameter estimate}}{95\%\text{CDIUB}}.$$

where 95% CDILB and 95% CDIUB refer to the lower and upper bounds for the 95% CDI based on the HDI.

A 95% CDI precision classification approach can also be applied in a similar manner to the RSE precision classification for the 95% CDIRAs. Here values ≥ 0.7 indicate high precision, 0.5–0.7 indicate moderate precision, 0.1–0.5 indicate low precision, and ≤ 0.1 indicate very low precision.

The code for population modeling performed in Stan as well as the 95% CDIRA implementation can be found in the Supporting Information S1.

## 4 Results

### 4.1 Sensitivity Analyses for PK and SOE Models

Figure 2 presents the results of the sensitivity analyses as well as the model predictions for the PK and SOE models respectively.

Both the PK and SOE models have similar prediction trajectories compared to the observed simulated plasma concentration data (Figure 2, C and D). For the PK model, the parameter CL has the highest Sobol indices over time (> 0.9) (Figure 2A), whereas the highest Sobol indices for the SOE model are with respect to the parameter $\beta_{1}$ with the highest value of 0.75. The parameters which have the lowest Sobol indices are $K_{A}$ and a for the PK and SOE models respectively.

### 4.2 Population Distributions for the PK Model

**FIGURE 3:**
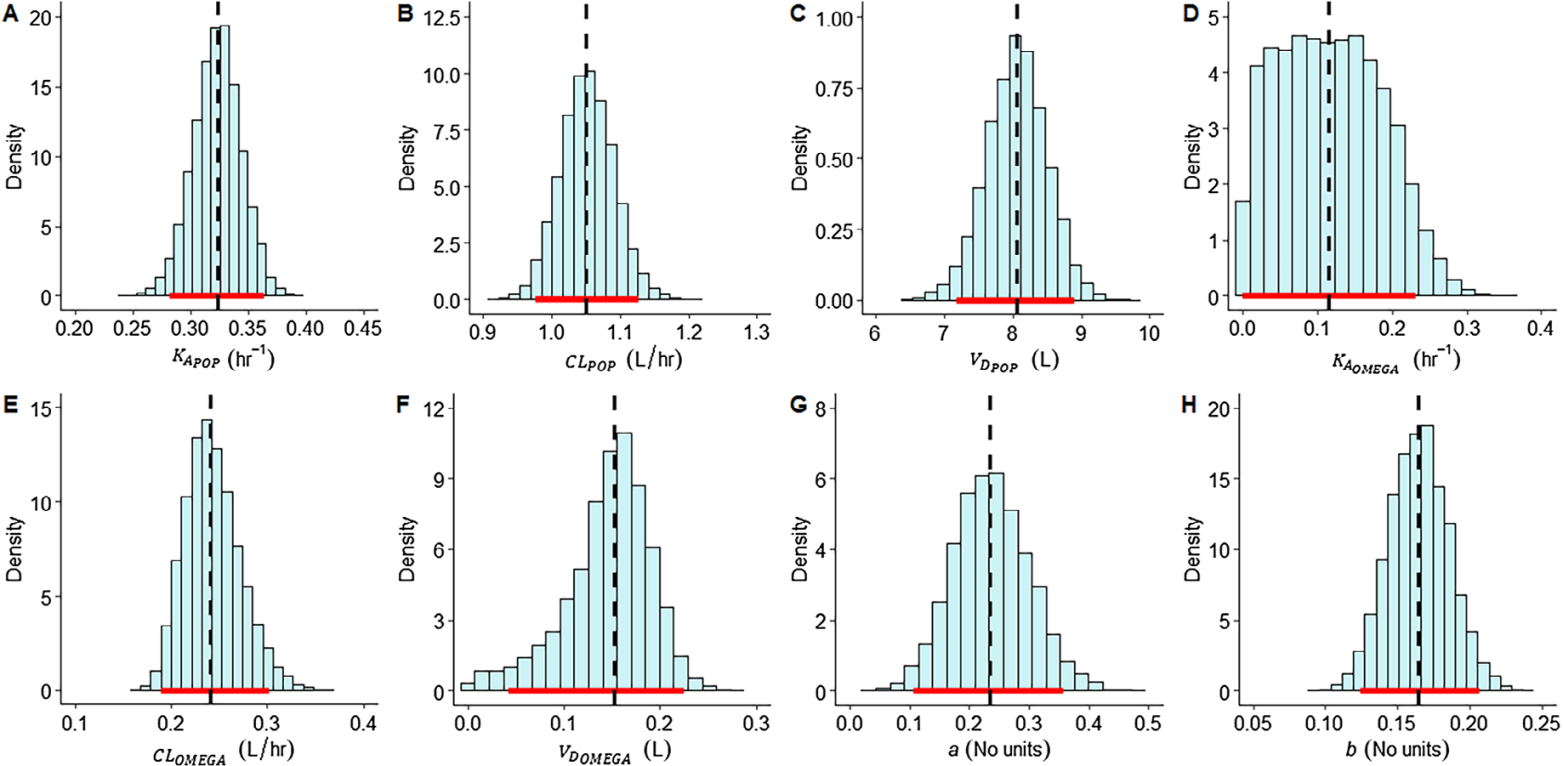
*P*opulation parameter distributions for the PK model. Density histograms for the population posterior parameter estimates for the PK model. Each histogram showcases the parameter estimates from the Hamiltonian Monte Carlo draws, their respective density values, the median estimate and the 95% credible interval.

Figure 3 shows the population parameter distributions for the PK model, using the simulated plasma concentration data.

The parameter, $K_{A_{\mathrm{POP}}}$, has the narrowest distribution with values between 0.241 and 0.394 h^−1^ (Figure 3A). In contrast the parameter, $K_{A_{\text{OMEGA}}}$, has the widest distribution with values between 2.470 $\times 10^{-5}$ and 0.358 h^−1^ (Figure 3D).

### 4.3 Population Distributions for the SOE Model

**FIGURE 4:**
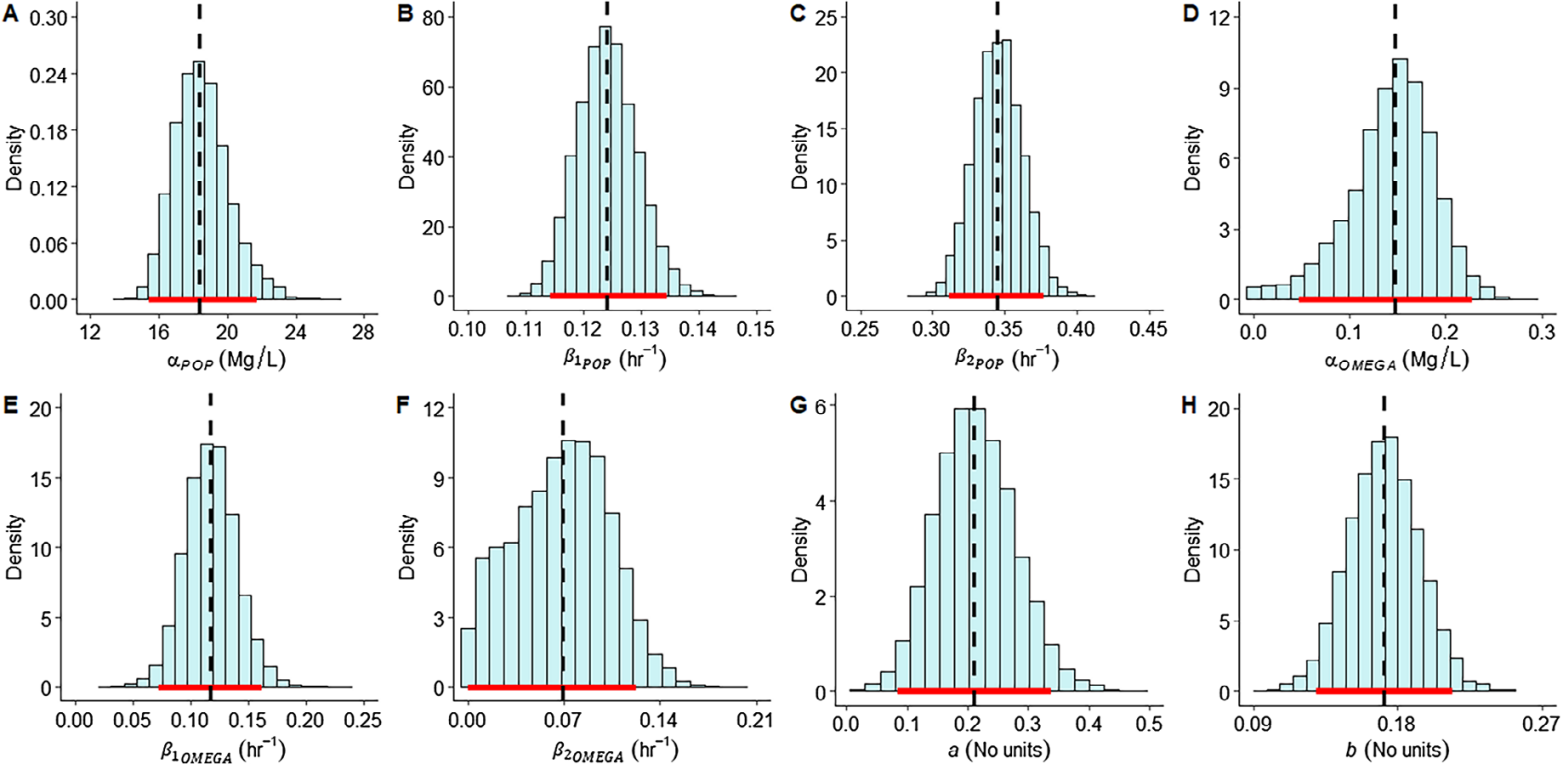
Population parameter distributions for the SOE model. Density histograms for the population posterior parameter estimates for the SOE model. Each histogram showcases the parameter estimates from the Hamiltonian Monte Carlo draws, their respective density values, the median estimate and the 95% credible interval.

Figure 4 shows the population parameter distributions for the SOE model, using the simulated plasma concentration data.

The parameter, $\beta_{1_{\mathrm{POP}}}$, has the narrowest distribution with values between 0.108 and 0.146 h^−1^. Whereas $\beta_{2_{\text{OMEGA}}}$, has the widest distribution with values between 5.057 $\times 10^{-5}$ and 0.199 h^−1^.

The individual parameter estimates and the random effects estimates can be found in the Supporting Information S1.

### 4.4 Uncertainty Analysis for the Population Estimates

Table 2 summarizes the UQ metrics for the parameter posterior distributions estimated in Stan.

On average the RSE values for the PK model are lower (19.652%) than those for the SOE model (20.200%). Similarly, the average 95% CDIRAs are higher for the PK model (0.622 (lower bound) and 0.768 (upper bound)) compared to those for the SOE model (0.595 (lower bound) and 0.755 (upper bound)). The RSE_CV for the parameter, $K_{A_{\text{OMEGA}}}$ is 59.713% whereas the 95% CDIRAs are 0.001 (lower bound) and 0.495 (upper bound). Likewise, the RSE_CV for the parameter $\beta_{2_{\text{OMEGA}}}$ is 49.869% whereas the 95% CDIRAs are 0.003 (lower bound) and 0.567 (upper bound).

### 4.5 HMC Diagnostic Measures

Table 3 summarizes the diagnostic values for the HMC chains for the population PK model.

During the initial estimation process, the standard deviations for the population values were set to one, yielding.

$\hat{R}$ values for the parameters $K_{A_{\mathrm{POP}}}$, $K_{A_{\text{OMEGA}}}$, $V_{D_{\mathrm{POP}}}$ and $V_{D_{\text{OMEGA}}}$ are > 1.1 and the associated ESS values < 400. However, when these standard deviations were set to the current model settings (Table 1), each of these parameters has ESS values > 400 and only the parameter $K_{A_{\text{OMEGA}}}$ has a value > 1.01 (1.012) (Table 3).

**TABLE 1:** Prior distributions summary.

	Distribution	Range
**PK model**		
$K_{A_{\mathrm{POP}}}$ (h^−1^)	Lognormal$\left(K_{A_{\text{Initial}\;\mathrm{pop}\;\text{value}}},0.3\right)$	≥ 0
${\mathrm{CL}}_{\mathrm{POP}}$ (L/h)	Lognormal(${\mathrm{CL}}_{\text{Initial}\;\mathrm{pop}\;\text{value}},0.5$)	≥ 0
$V_{D_{\mathrm{POP}}}$ (L)	Lognormal$\left(V_{D_{\text{Initial}\;\mathrm{pop}\;\text{value}}},0.5\right)$	≥ 0
$K_{A_{\text{OMEGA}}}$ (h^−1^)	Normal(0, 0.5)	≥ 0
${\mathrm{CL}}_{\text{OMEGA}}$ (L/h)	Normal(0, 0.5)	≥ 0
$V_{D_{\text{OMEGA}}}$ (L)	Normal(0,0.5)	≥ 0
$\;K_{A_{\mathrm{\eta i}}}$ (h^−1^)	Normal(0,1)	No limits
${\mathrm{CL}}_{\mathrm{\eta i}}$ (L/h)	Normal(0,1)	No limits
$\;V_{D_{\mathrm{\eta i}}}$ (L)	Normal(0,1)	No limits
a (No units)	Normal(0,1)	≥ 0
b (No units)	Normal(0,0.3)	≥ 0
**SOE model**		
$\alpha_{\mathrm{POP}}$ (Mg/L)	Lognormal($\alpha_{\text{Initial}\;\mathrm{pop}\;\text{value}},0.5$)	≥ 0
$\beta_{1_{\mathrm{POP}}}$ (h^−1^)	Lognormal$\left(\beta_{1_{\text{Initial}\;\mathrm{pop}\;\text{value}}},0.5\right)$	≥ 0
$\beta_{2_{\mathrm{POP}}}$ (h^−1^)	Lognormal$\left(\beta_{2_{\text{Initial}\;\mathrm{pop}\;\text{value}}},0.5\right)$	≥ 0
$\alpha_{\text{OMEGA}}$ (Mg/L)	Normal(0, 1)	≥ 0
$\beta_{1_{\text{OMEGA}}}$ (h^−1^)	Normal(0,1)	≥ 0
$\beta_{2_{\text{OMEGA}}}$ (h^−1^)	Normal(0,1)	≥ 0
$\alpha_{\mathrm{\eta i}}$ (Mg/L)	Normal(0,1)	No limits
$\beta_{1_{\mathrm{\eta i}}}$ (h^−1^)	Normal(0, 1)	No limits
$\beta_{2_{\mathrm{\eta i}}}$ (h^−1^)	Normal(0, 1)	No limits
a (No units)	Normal(0,1)	≥ 0
b (No units)	Normal(0,0.3)	≥ 0

*Note:* Summary of the prior parameter distributions used in the model. For the model parameters, the term “POP” refers to the typical parameter estimate, “OMEGA” refers to the between subject variation (BSV) and “i” refers to individuals. The parameters *a* and *b* are the proportional error model parameters. For the distributions, the numbers in the brackets refer to the mean and standard deviation. Lastly the limits refer to any boundaries applied to the parameter estimates.

## 5 Discussion

The sensitivity analysis already indicates that, on average, the predicted plasma concentration model output is more sensitive to the PK model than the reparameterized SOE model (Figure 2). Although the parameter CL has the highest Sobol indices (Figure 2A), there is a steep decline in the Sobol indices for the parameter $K_{A}$ during the first 3 h of the plasma concentration profile which may suggest that it is more feasible to estimate this parameter during the first few hours after the dose has been administered. The parameter $\beta_{2}$ which represents the parameter $K_{A}$ in the SOE model (Equations 4 and 5), does not follow this trend (Figure 2A,B). This may be due to parameter α, which is estimated to take into account the absorption magnitude and given that this parameter also incorporates the dose and all of the other parameters.

Both the PK and the SOE models produce similar predictive fits to the observed data (Figure 2C,D). This further demonstrates that even though reparameterized (data‐driven) models may still fit similarly to the data, there may still be differences associated with model/parameter sensitivities and epistemic uncertainties.

The posterior distributions provide a visual uncertainty summary of each of the parameter estimates. The posterior distribution for the parameter $K_{A_{\text{OMEGA}}}$, has highest uncertainty (Figure 3D). The minimum value is 2.47 $\times 10^{-5}$ and the 95% CDI is 6.413 $\times 10^{-5}$ which suggest that it is highly credible that the estimated BSV may be zero and that there is not enough knowledge from both the data and model to identify that there is BSV (Table 2). This also applies to the parameter $\beta_{2_{\text{OMEGA}}}$ (Figure 4F) which is the parameter $K_{A_{\text{OMEGA}}}$ in the reparameterized SOE model. While the parameter $K_{A_{\mathrm{POP}}}$ has the narrowest distribution for the PK model and the parameter $\beta_{1_{\mathrm{POP}}}$ (Figure 4B) which is the reparameterized $K_{E_{\mathrm{POP}}}$ (${\mathrm{CL}}_{\mathrm{POP}}$/$V_{D_{\mathrm{POP}}}$), has the narrowest distribution for the SOE model, the second narrowest distribution for both the PK and SOE models are ${\mathrm{CL}}_{\mathrm{POP}}$ (Figure 3B) and $\beta_{2_{\mathrm{POP}}}$ (Figure 4C) respectively. The combined error model parameter distributions have similar profiles for both the PK (Figure 3G,H) and SOE models (Figure 4G,H). Given that both models use the same data and the error model parameter estimates are similar, most of the uncertainties can be attributed to epistemic uncertainties.

**TABLE 2:** UQ metrics for the parameter posterior distributions.

	Median	RSE_CV(%)	95% CDI	95% CDIRALB	95% CDIRAUB
**PK model**					
$K_{A_{\mathrm{POP}}}$ (h^−1^)	0.323	6.433	0.283–0.364	0.874	0.888
${\mathrm{CL}}_{\mathrm{POP}}$ (L/h)	1.051	3.707	0.976–1.128	0.929	0.932
$V_{D_{\mathrm{POP}}}$ (L)	8.053	5.478	7.175–8.898	0.891	0.905
$K_{A_{\text{OMEGA}}}$ (h^−1^)	0.115	59.713	6.413 × 10^−5^–0.232	0.001	0.495
${\mathrm{CL}}_{\text{OMEGA}}$ (L/h)	0.241	12.083	0.190–0.302	0.791	0.796
$V_{D_{\text{OMEGA}}}$ (L)	0.152	29.767	0.043–0.225	0.283	0.676
a (No units)	0.234	27.190	0.107–0.358	0.456	0.655
b (No units)	0.165	12.848	0.124–0.207	0.755	0.794
**SOE model**					
$\alpha_{\mathrm{POP}}$ (Mg/L)	18.367	8.802	15.401–21.731	0.839	0.845
$\beta_{1_{\mathrm{POP}}}$ (h^−1^)	0.124	4.192	0.114–0.134	0.921	0.922
$\beta_{2_{\mathrm{POP}}}$ (h^−1^)	0.345	4.865	0.312–0.377	0.904	0.915
$\alpha_{\text{OMEGA}}$ (Mg/L)	0.148	30.486	0.047–0.228	0.319	0.648
$\beta_{1_{\text{OMEGA}}}$ (h^−1^)	0.117	19.274	0.073–0.162	0.621	0.723
$\beta_{2_{\text{OMEGA}}}$ (h^−1^)	0.069	49.869	1.780 × 10^−4^–0.123	0.003	0.567
a (No units)	0.210	31.211	0.083–0.337	0.398	0.623
b (No units)	0.171	12.901	0.129–0.215	0.754	0.799

*Note:* A summary of the median, RSE, 95% CDI and 95% CDIRA values for the PK and SOE models.

Abbreviations: 95% CDI, 95% credible intervals; 95% CDIRALB, 95% credible interval average lower bound; 95% CDIRAUB, 95% credible interval average upper bound; RSE_CV, Relative standard error based on the covariance matrix.

The parameters $K_{A_{\mathrm{POP}}}$ and $\beta_{2_{\mathrm{POP}}}$ have similar median values and 95% CDIs. Likewise, the parameter $\beta_{1_{\mathrm{POP}}}$ and the parameter combination ${\mathrm{CL}}_{\mathrm{POP}}$/$V_{D_{\mathrm{POP}}}$ also share this relationship (Table 2). On average the SOE model has the highest uncertainties which suggest that the PK model is a more credible fit to the data. In Table 2, both the RSE and the 95% CDIRAs highlight which parameter estimates have the lowest UQ measures ($K_{A_{\text{OMEGA}}}$ for the PK model and $\beta_{2_{\text{OMEGA}}}$ for the SOE model). Interestingly, while both metrics agree on those parameters that have high precision (e.g., CL_POP_ (RSE: 3.707% and CDIRAs: 0.929 (Lower bound) and 0.932 (upper bound))), the precision classification for the less credible parameters are different. In Table 2, the parameter $\beta_{2_{\text{OMEGA}}}$ has an RSE of 49.869% which suggests moderate precision. This classification aligns with the 95% CDIRAUB (0.567), however the 95% CDIRALB is 0.003 which suggests very low precision. The 95% CDIRA classifications align more with the posterior distribution and thus the uncertainty of the parameter $\beta_{2_{\text{OMEGA}}}$. As the RSE is based primarily on variation, which uses the summation of all the data points compared to the mean, higher values will mask the lower values and thus overestimate the result in terms of precision. In contrast, the 95% CDIRAs include both lower and upper bound metrics which are already based on the 95% CDI and highlight how far the data points are in each direction with respect to the typical parameter estimate (Figure 1).

Additionally, the RSE classification levels also underestimate the diagnostic issues that arise during the HMC processes. Table 3 highlights the $\hat{R}$ values and the ESS_Bulk and ESS_Tail values associated with the PK model when the initial standard deviation used for the population parameter estimates was set to one compared to the values defined in Table 1. The high $\hat{R}$ values, along with the low ESS values, indicate low convergence and unreliable estimates. While higher RSE values are associated with the parameters $K_{A_{\mathrm{POP}}}$ (26.297%), $K_{A_{\text{OMEGA}}}$ (65.207%), $V_{D_{\mathrm{POP}}}$ (26.163%) and $V_{D_{\text{OMEGA}}}$ (34.058%) it is only the parameter $K_{A_{\text{OMEGA}}}$ which is classed as low precision whereas the other parameters are classed as high precision. In contrast with the 95% CDIRAs the lower bound ratios show that the highest classification is low precision for these parameter, with $\hat{R}$ value above 1.01 (with the parameter $K_{A_{\text{OMEGA}}}$ classed as very low precision). The high $\hat{R}$, ESS_Bulk and ESS_Tail values indicate poor convergence and sampling, potentially leading to multimodal distributions and incomplete exploration of the parameter space. These issues impacts the density and thus the 95% CDI. In contrast, the RSE which only considers the variance of the distribution underestimates the risk of diagnostic issues, which would indicate more uncertainty surrounding parameter estimates.

**TABLE 3:** Diagnostic values for population PK parameter posterior distributions.

	Median	$\hat{R}$	ESS_Bulk	ESS_Tail	RSE_CV(%)	95% CDIRALB	95% CDIRAUB
**PK model (Standard deviation was set to 1 for population estimates)**							
$K_{A_{\mathrm{POP}}}$ (h^−1^)	0.294	1.530	7.299	27.345	26.297	0.413	0.867
${\mathrm{CL}}_{\mathrm{POP}}$ (L/h)	1.056	1.003	940.906	1535.280	3.709	0.929	0.933
$V_{D_{\mathrm{POP}}}$ (L)	7.486	1.531	7.291	30.735	26.163	0.394	0.885
$K_{A_{\text{OMEGA}}}$ (h^−1^)	0.115	1.244	14.140	87.024	65.207	0.004	0.483
${\mathrm{CL}}_{\text{OMEGA}}$ (L/h)	0.239	1.026	293.271	1952.735	12.374	0.785	0.798
$V_{D_{\text{OMEGA}}}$ (L)	0.148	1.111	30.973	173.197	34.058	0.185	0.649
a (No units)	0.240	1.003	2305.128	2779.901	25.758	0.486	0.672
b (No units)	0.162	1.002	2274.771	2515.835	12.599	0.748	0.807
**PK model (Standard deviation was set to the values in Table** **1** **for the population estimates)**							
$K_{A_{\mathrm{POP}}}$ (h^−1^)	0.323	1.001	2471.450	2669.881	6.433	0.874	0.888
${\mathrm{CL}}_{\mathrm{POP}}$ (L/h)	1.051	1.001	1352.991	2700.400	3.707	0.929	0.932
$V_{D_{\mathrm{POP}}}$ (L)	8.053	1.001	1849.730	2914.117	5.478	0.891	0.905
$K_{A_{\text{OMEGA}}}$ (h^−1^)	0.115	1.012	560.924	1487.381	59.713	0.001	0.495
${\mathrm{CL}}_{\text{OMEGA}}$ (L/h)	0.241	1.003	1977.684	3278.239	12.083	0.791	0.796
$V_{D_{\text{OMEGA}}}$ (L)	0.152	1.006	610.397	630.610	29.767	0.283	0.676
a (No units)	0.234	1.001	4028.909	4152.827	27.190	0.456	0.655
b (No units)	0.165	1.001	4315.200	4442.366	12.848	0.755	0.794

*Note:* Summary of diagnostic values for PK parameter posterior distributions when the standard deviations were set to 1 for the population estimates versus the current settings in Table 1.

Abbreviations: 95% CDI, 95% credible intervals; 95% CDIRALB, 95% credible interval average lower bound; 95% CDIRAUB, 95% credible interval average upper bound; ESS_Bulk, Effective sample size bulk and ESS_Tail, Effective sample size tail, both of which measures the sampling efficiency within the distribution; $\hat{R}$, R‐hat value which measures between and within chain variance; RSE_CV, Relative standard error based on the covariance matrix.

An important note is that while typical PKPD modeling software also includes the provision of 95% confidence intervals, the derivations of these intervals are typically based on variance or bootstrapping methods. These approaches do not reflect the overall uncertainty of a parameter.

While the 95% CDIRAs are a useful UQ metric, it is important to consider associated limitations. Firstly, this metric is dependent on a posterior distribution which many PKPD modeling software do not primarily employ as a method for parameter estimation. In this study the estimation process was performed in Stan where the average computation time was 10 min for the population PK model and less than 3 min for the population SOE model. This contrasts with running both models in Monolix which takes less than 1 min to estimate the parameters for both models. For more complex models and sparse datasets the computation time in Stan may significantly increase. While the Supporting information provides the code necessary for modelers to build and estimate their population models in Stan, the reality is that a more “hands‐on” approach is needed in terms of tuning the HMC algorithm and the prior model distributions whereas in typical PKPD modeling software it is only the distributions and error models that are altered. Additionally, the 95% CDIRA range of 0–1, does not hold for negative parameter estimates. However, the equations can be updated to use absolute values in the case of negative estimates. Further work will involve investigating UQ techniques for data‐driven approaches as, even though UQ implementation is demanding, it is necessary for credible and reliable model informed drug development.

To conclude this study, both the RSE and 95% CDIRAs showcase that the PK model is more credible and precise than the reparameterized SOE model for the case study considered here. However, it is through analysis of the 95% CDIRAs that the uncertainties can be highlighted and robust precision results, particularly for asymmetrical distributions, can be determined. This aspect is ignored in RSE calculations, which primarily only rely on the variance of the distribution. Further work will consider wider UQ implementation (using question centric approaches) across different stages in the model development process, as well as UQ metrics for data‐driven approaches.

## Author Contributions

L.W., I.S.R., J.K., and M.J.C. wrote the manuscript; L.W., I.S.R., J.K., and M.J.C. designed the research; L.W. performed the research; L.W., J.K., and M.J.C. analyzed the data.

## Funding

This research is funded by the UK Research and Innovation (grant number: 10106221) and the European Union. Views and opinions expressed are, however, those of the author(s) only and do not necessarily reflect those of the European Union or the European Health and Digital Executive Agency (HaDEA). Neither the European Union nor the granting authority can be held responsible for them.

## Conflicts of Interest

The authors declare no conflicts of interest.

## Supporting information


**Table S1:** Profile likelihood analysis for PK model population parameters; $K_{A_{\mathrm{POP}}}$, ${\mathrm{CL}}_{\mathrm{POP}}$ and $V_{D_{\mathrm{POP}}}$. CI refers to the profile likelihood based confidence intervals. The threshold value is 1567.994 for all of the parameters, where $L\left(p_{\text{median}}\right)$ is −1566.073.
**Table S2:** Profile likelihood plots for SOE model population parameters $\alpha_{\mathrm{POP}}$, $\beta_{1_{\mathrm{POP}}}$ and $\beta_{2_{\mathrm{POP}}}$. The threshold value for each of these parameters is 1039.444, where $L\left(p_{\text{median}}\right)$ is −1039.444.
**Table S3:** Individual parameters and random effects estimate for PK model.
**Table S4:** Individual parameters and random effects estimate for reparameterized SOE model.
