# Supplementary material for: The 95% CDIRAs, a Credible Interval Based Method to Capture Uncertainty in Population Modeling: Pharmacokinetics Versus Sum of Exponentials Case Study
Source: CPT Pharmacometrics Syst Pharmacol. 2026 Aug 27;15(9):e70326. doi: 10.1002/psp4.70326 (PMC13519695; doi:10.1002/psp4.70326)
Supplement: Supplementary file 1 [file PSP4-15-e70326-s001.docx]

Supplementary materials

Sensitivity analysis

Sensitivity analysis explores the relationship between the model inputs and outputs. The effects of altering a particular model parameter have on a model output can be assessed and use to determine if a particular output is sensitive to a particular parameter. A parameter can be deemed as sensitive if changing the value of a particular parameter slightly has a significant effect on the model outputs. similarly, a parameter may be deemed as insensitive if significantly changing a particular parameter value does not impact the model output. Moreover, if changes the parameter value significantly does not cause any impact on a model output, this may indicate that the parameter is unidentifiable (a structural identifiability analysis would be needed to confirm this assumption) and/or may be unnecessary for the model. The sensitivity changes also enable modellers to confirm if the modelled system in accordance with the represented system.

In the main article, the Sobol indices method was used to assess the sensitivity analysis for both the pharmacokinetic (PK) model and the sum of exponentials (SOE) model. The Sobol indices essentially categorise the variances of a model’s output (the response) based on the model’s inputs (i.e. the parameters and other states that are involved in the system). Model’s inputs which produce higher variances are more sensitive compared to inputs which produce low variances.

Structural identifiability analysis

As mentioned briefly in the previous section, a model parameter that has no impact on a model output may indicate identifiability issues. However structural identifiability analysis (SIA) is required to confirm this assumption. SIA assesses whether based on a model’s input(s) output(s) and initial conditions if for a particular model parameter *p_i_*, only a finite set of values or an infinite set of values can be used to generate the same exact model output (*y*(*t*)). If *p_i_* can be uniquely determined from *y*(*t*), then *p_i_* is classed as globally identifiable. If there exists a neighbourhood from which *p_i_* can be identified, then *p_i_* is said to be locally identifiable. However, if *p_i_* cannot be identified globally or locally and there exists infinite values which can be used to identify *p_i_* and lead to the same *y*(*t*), then *p_i_* is structurally unidentifiable. For a model to be classed as structurally identifiable all the parameters must be classed as at-least locally identifiable. If any parameter is structurally unidentifiable then any predictions based on this model without any modifications is unreliable as if any value can be used to generate the same output then the likelihood of a particular parameter value being correct is very minimal.

There are many methods which can be used to assess the SIA of the model, in this article the Taylor series approach is chosen for its simplicity and applicability to both linear and nonlinear models as well as both fixed effects and mixed effects model. This article will only focus on the structural identifiability for the base model to keep this guide relatively simple however the concepts can be applied to mixed effects model also.

The Taylor series expansion is as follows:

$f\left( x \right)=f\left( a \right)+f^{'}\left( a \right)\left( x-a \right)+\frac{f^{''}(a)}{2!}{(x-a)}^{2}\ldots+\frac{f^{n}(a)}{n!}{(x-a)}^{n}$

where *f*(*x*) is the output function (*y*(*t*)) and *a* is point at which the function is evaluated. For the SIA, this time point is usually set to 0, as this is often a known time point for the output. An interesting feature of the Taylor series is that all the derivatives from the observed *y*(*t*), which form the coefficients of this series are assumed to be unique and measurable and therefore known. The solutions of these derivatives can therefore be assessed to determine if the model parameters can be identified for SIA. The next two sections cover the SIA using the Taylor series approach for both the PK and SOE model

PK model SIA

To recap, the system of ODEs which are used to describe the PK model is described below:

$\frac{d(Depot)}{dt}= {-K}_{A}\cdot Depot(t)$	(1)

$\frac{d(Central)}{dt}= K_{A}\cdot Depot\left( t \right)-\frac{CL}{V_{D}}\cdot Central(t)$	(2)

$y\left( t \right)= \frac{Central(t)}{V_{D}}$	(3)

Where the initial conditions for the *Depot* and *Central* are 100mg and 0mg, respectively.

The first three derivatives for the observed output ($plasma\left( t \right)= \frac{Central(t)}{V_{D}}$) at time *t* are as follows:

$Plasma^{'}\left( t \right)= \frac{K_{A}\cdot V_{D}\cdot Depot\left( t \right)-CL\cdot Central(t)}{V_{D}^{2}}$	(S1)

$Plasma^{''}\left( t \right)= \frac{K_{A}\cdot V_{D}\cdot(CL+K_{A}\cdot V_{D})\cdot Depot\left( t \right)-{CL}^{2}\cdot Central(t)}{V_{D}^{3}}$	(S2)

$Plasma^{'i'}\left( t \right)= \frac{K_{A}\cdot V_{D}\cdot({CL}^{2}+K_{A}\cdot V_{D}\cdot\left( CL+K_{A}\cdot V_{D} \right))\cdot Depot\left( t \right)-{CL}^{3}\cdot Central(t)}{V_{D}^{4}}$	(S3)

The first three derivatives for the observed output when *t* = 0 are as follows:

$Plasma^{'}\left( 0 \right)= \frac{{100\cdot K}_{A}}{V_{D}}$	(S4)

$Plasma^{''}\left( 0 \right)= \frac{100\cdot K_{A}\cdot(CL+K_{A}\cdot V_{D})}{V_{D}^{2}}$	(S5)

$Plasma^{'''}\left( 0 \right)= \frac{100\cdot K_{A}\cdot({CL}^{2}+K_{A}\cdot V_{D}\left( CL+K_{A}\cdot V_{D} \right))}{V_{D}^{3}}$	(S6)

Note that *Depot*(0) is the dose which is 100mg. Eqns(S4 – S6) can be used to identify all the parameters for the PK model. The steps of which are described below:

1) Eqn(S4) can be used to solve for $V_{D}$ :

$V_{D}=\frac{100\cdot K_{A}}{Plasma^{'}\left( 0 \right)}$

2) Substitute new solution for $V_{D}$ into Eqn(S5)

$Plasma^{''}\left( 0 \right)= -\frac{CL\cdot{Plasma^{'}\left( 0 \right)}^{2}}{100\cdot K_{A}}-Plasma^{'}\left( 0 \right)\cdot K_{A}$

3) Use new equation in step 2 to solve for *CL*:

$CL=-\frac{100\cdot K_{A}\cdot(Plasma^{''}\left( 0 \right)+Plasma'(0)\cdot K_{A})}{{Plasma^{'}\left( 0 \right)}^{2}}$

4) Substitute both new solutions for $V_{D}$ and *CL* into Eqn(S6):

$Plasma^{'''}\left( 0 \right)= \frac{{Plasma^{''}\left( 0 \right)}^{2}}{Plasma'(0)}+Plasma^{''}\left( 0 \right)\cdot K_{A}+Plasma'(0)\cdot K_{A}^{2}$

5) Since the *plasma* derivates at time *0* are assumed to be known and are unique the only unknown parameter is $K_{A}$, therefore $K_{A}$ can be identified from step 4
6) This subsequently means that *CL* can be identified from step 2 and $V_{D}$ can be identified from step 1
7) All the parameters have been identified and therefore the PK model is structurally identifiable

SOE model SIA

The SOE model used to represent the simulated plasma concentration is as follows:

$Plasma\left( t \right)= \alpha\cdot e^{-\beta_{1}\cdot t}-\alpha\cdot e^{-\beta_{2}\cdot t}$	(4)

The initial condition is 0mg. The first three derivatives for *Plasma*(*t*) using the SOE model are as follows:

$Plasma^{'}\left( t \right)= -\alpha\cdot\beta_{1}\cdot e^{-\beta_{1}\cdot t}+\alpha\cdot\beta_{2}\cdot e^{-\beta_{2}\cdot t}$	(S7)

$Plasma^{''}\left( t \right)= \alpha\cdot{\beta_{1}}^{2}\cdot e^{-\beta_{1}\cdot t}-\alpha\cdot{\beta_{2}}^{2}\cdot e^{-\beta_{2}\cdot t}$	(S8)

$Plasma^{'''}\left( t \right)= -\alpha\cdot{\beta_{1}}^{3}\cdot e^{-\beta_{1}\cdot t}+\alpha\cdot{\beta_{2}}^{3}\cdot e^{-\beta_{2}\cdot t}$	(S9)

The first three derivatives for the observed plasma concentration when *t* = 0 are as follows:

$Plasma^{'}\left( 0 \right)= -\alpha\cdot\beta_{1}+\alpha\cdot\beta_{2}$	(S10)

$Plasma^{''}\left( 0 \right)= \alpha\cdot{\beta_{1}}^{2}-\alpha\cdot{\beta_{2}}^{2}$	(S11)

$Plasma^{'''}\left( 0 \right)= -\alpha\cdot{\beta_{1}}^{3}+\alpha\cdot{\beta_{2}}^{3}$	(S12)

The following steps can be taken to assess the SIA of the SOE model:

1) Use Eqn(S10) to solve for $\alpha$:

$\alpha=\frac{Plasma^{'}\left( 0 \right)}{-\beta_{1}+\beta_{2}}$

2) Substitute new solution for $\alpha$ into Eqn(S11):

$Plasma^{''}\left( 0 \right)=-((\beta_{1}+\beta_{2})\cdot Plasma'(0)$

3) Use step two to solve for $\beta_{1}$ :

$\beta_{1}=-\frac{\beta_{2}\cdot Plasma^{'}\left( 0 \right)+Plasma''(0)}{Plasma'(0)}$

4) Substitute the new solutions for $\alpha$ and $\beta_{1}$ into Eqn(S12):

$Plasma^{'''}\left( 0 \right)={\beta_{2}}^{2}\cdot Plasma^{'}\left( 0 \right)+\beta_{2}\cdot Plasma^{''}\left( 0 \right)+\frac{{Plasma^{''}\left( 0 \right)}^{2}}{Plasma^{'}\left( 0 \right)}$

5) The only unknown parameter in step 4 is $\beta_{2}$ and therefore $\beta_{2}$ can be identified
6) Given that $\beta_{2}$ is now identifiable, $\beta_{1}$ can now be identified from step 3
7) Lastly, $\alpha$ can be identified from step 1 which means that all parameters have been identified for the model and the model is now deemed as structurally identifiable

Practical identifiability analysis

The concept of SIA is extended in practical identifiability analysis (PIA) to assess whether based on the model system and the associated data if all the unknown parameters can be identified. A parameter is classed as practically identifiable if there is enough evidence to indicate that changing the parameter values has a significant impact on the likelihood of the observed response. There are multiple methods to assess PIA, in this study the profile likelihood approach was chosen due to the simplicity of the application.

To assess whether a particular parameter, *p_i_* is practically identifiable under the profile likelihood approach, a profile which summarises the different likelihood values for discretised values of *p_i_* is generated. These discretised values are usually centred around the optimal value for *p_i_*. For each discretised value of *p_i_*, the other parameters in the model are reoptimised to obtain the best likelihood value based on the data and the model. The profile likelihood approach utilise a profile likelihood based confidence interval threshold to assess whether there is enough evidence to suggest that the changes in the likelihood values for the model and the data based on the changes in the *p_i_* values is significant enough for the parameter to be deemed as practically identifiable. The threshold is usually based on the maximum likelihood estimate (MLE). However, given that the median parameter values were extracted, in this study the threshold was based on the likelihood of the median parameter value:

$Threshold=L\left( p_{median} \right)+\frac{x^{2}(0.95,1)}{2}$	(S13)

where $L\left( p_{median} \right)$ refers to the likelihood of the mean value for the parameter in other words the likelihood for the pop parameters. Both the lower values as well as the upper values of *p_i_* are required to pass this threshold for the parameter to be deemed as practically identifiable. The points at which the profile likelihood curve crosses this threshold are equivalent to the 95% profile likelihood-based confidence intervals. In this analysis, the step size which governs the discretised values for *p_i_* is set to 0.01. This average value for the interval ranges that were supplied for each of the parameters (0.0001 to 100). The profile likelihood tables the typical population parameters for both the PK and SOE model are provided in Tables S1 and S2

Practical identifiability analysis for PK model

| Parameters | 95% CI lower bound | 95% CI upper bound |
| --- | --- | --- |
| ${K_{A}}_{POP}$ (hr^-1^) | 0.060 | 0.333 |
| ${CL}_{POP}$ (L/hr) | 0.900 | 2.451 |
| ${V_{D}}_{POP}$ (L) | 3.800 | 8.753 |

Table S1: profile likelihood analysis for PK model population parameters; ${K_{A}}_{POP}$ , ${CL}_{POP}$ and ${V_{D}}_{POP}$. CI refers to the profile likelihood based confidence intervals. The threshold value is 1567.994 for all of the parameters, where $L\left( p_{median} \right)$ is -1566.073

All of the PK population parameters cross the threshold at the lower and upper 95% CI bounds. Therefore, for each parameter there is enough evidence to suggest that variations in both the lower and upper values have a significant effect on the resulting likelihood and therefore all these parameters are practically identifiable.

Practical identifiability analysis for SOE model

| Parameters | 95% CI lower bound | 95% CI upper bound |
| --- | --- | --- |
| $\alpha_{POP}$ (Mg/L) | 18.20 | 22.967 |
| ${\beta_{1}}_{POP}$ (hr^-1^) | 0.110 | 0.144 |
| ${\beta_{2}}_{POP}$ (hr^-1^) | 0.280 | 0.395 |

Table S2: Profile likelihood plots for SOE model population parameters $\alpha_{POP}$, ${\beta_{1}}_{POP}$ and ${\beta_{2}}_{POP}$. The threshold value for each of these parameters is 1039.444, where $L\left( p_{median} \right)$ is -1039.444

Similar to the PK model, the reparameterised SOE model is also practically identifiable as all parameters cross the threshold at both the 95% CI lower and upper bounds. Interestingly, the likelihood for the reparameterised model is also lower than the PK model which would suggest that this provides a better prediction fit to the data.

Model codes

The packages in the *library* command need to be installed prior to script use. Most of these packages can be installed in *R* using the command *install.packages(“package name”)*. To install the package *cmdstanr* please refer to the guidelines here: <https://mc-stan.org/cmdstanr/articles/cmd>.

It is expected that users who wish to use these scripts have access to the *Monolix* Oral1 dataset. In the scripts, the downloaded PK dataset is named as *MonolixPKData* (this data should be a data frame with four columns: id,time,amt,y).

The model codes provide the Stan scripts for the development of the population PK and SOE models, extraction of parameter estimates, standard error values and 95% CDIRA calculations. Note that lines which start with “#” or “//” are comments to help the users understand the script.

Population PK model

\### Commencement of script

library(rstan)

library(bayestestR)

library(posterior)

library(readxl)

library(dplyr)

library(tidyr)

library(ggplot2)

library(ggpubr)

library(HDInterval)

library(deSolve)

library(cmdstanr)

\# Converting the *Monolix* data to a matrix format that can be implemented into *Stan*

\# This matrix will now have 11 rows (which represents the number of timepoints) and 50 columns (which represents the number of patients)

pkdata<-matrix(as.vector(unlist(MonolixPKData$y)),11,50)

##Stan model

\# Cmdstan requires for the STAN model to be written and then compiled

\# Typical writing blocks are data (type of data that will be used in the model), parameters (parameters to estimate, the dimensions and if any restrictions should be in place) and model (distributions for the parameters as well as the model for the observation).

\# As an ODE solver is needed, there is a function section to describe the ODE system

#An additional block (Generating quantities) is included to obtain the model likelihood values

MixedEffectsPK_model="

functions { //Defining the ODEs and the data type in the ODE system

vector sho(real t,

vector y,

real KA_POP,

real CL_POP,

real V_POP) {

vector[2] dydt;

dydt[1] = -KA_POP*y[1];

dydt[2] = KA_POP*y[1]-(CL_POP/V_POP)*y[2];

return dydt;

}

}

data {

int<lower=1> T;

int<lower=1> N_ID;

matrix[N_ID,T] y1;

vector[2] y0; // initial value for each of the states at the initial time since there are two observations this is a vector of 2

array[T] real ts;

real KA; //initial estimates and SDs (1 is often used as the typical SD)

real CL;

real V;

real t0;

}

parameters {

real<lower=0> KA_POP;

real<lower=0> CL_POP;

real<lower=0> V_POP;

real <lower=0> KA_OMEGA; //To make these distributions half-normal

real <lower=0> CL_OMEGA;

real <lower=0> V_OMEGA;

real <lower=0> a; //

real <lower=0> b; //

vector[N_ID] KA_nv; // the eta values for estimating the individual parameter estimates

vector[N_ID] CL_nv;

vector[N_ID] V_nv;

}

transformed parameters {

vector <lower=0> [N_ID] KA_IND;

vector <lower=0>[N_ID] CL_IND;

vector <lower=0>[N_ID] V_IND;

vector[N_ID] KA_ETA;

vector[N_ID] CL_ETA;

vector[N_ID] V_ETA;

KA_ETA = KA_OMEGA * KA_nv;

CL_ETA = CL_OMEGA * CL_nv;

V_ETA = V_OMEGA * V_nv;

for(i in 1:N_ID) {

KA_IND[i]=KA_POP*exp(KA_ETA[i]);

CL_IND[i]=CL_POP*exp(CL_ETA[i]);

V_IND[i]=V_POP*exp(V_ETA[i]);

}

}

model {

KA_POP ~ lognormal(log(KA),0.3);

CL_POP ~ lognormal(log(CL),0.5);

V_POP ~ lognormal(log(V),0.5);

KA_OMEGA~ normal(0,0.5);

CL_OMEGA~ normal(0,0.5);

V_OMEGA ~ normal(0,0.5);

a ~ normal(0,1);

b ~ normal(0,0.3);

for(i in 1:N_ID) {

KA_nv[i] ~ normal(0,1);

CL_nv[i] ~ normal(0,1);

V_nv[i] ~ normal(0,1);

array[T] vector[2] model1 = ode_rk45(sho, y0, t0, ts,KA_IND[i], CL_IND[i], V_IND[i]);

for (t in 1:T) {

y1[i,t] ~ normal((model1[t,2]/V_IND[i]),(a+b*((model1[t,2]/V_IND[i]))));

}

}

}

"

\## Converting the model into a cmdstan model

MixedEffectsPKmodel<-write_stan_file(MixedEffectsPK_model)

MixedEffectsPKmodel<-cmdstan_model(MixedEffectsPKmodel)

\## Data for the cmdstan model: everything that was declared in the data section needs to be included

MixedEffectsPKdata<-list(T=length(unique(Time)),y1=as.matrix(t(pkdata)),t0=0,y0=c(100,0),ts=unique(Time),KA=1,

CL=1,V=10,N_ID=50)# The parameters (KA,CL and V) are defined by the user, feel free to change them

\## Hamiltonian Monte Carlo sampling

##Note there may be some metropolis proposal rejections but these are quite rare,

MixedEffectsPKsamplefits <- MixedEffectsPKmodel$sample(

data = MixedEffectsPKdata,

chains = 4,

parallel_chains = 4,

iter_warmup = 1500,

iter_sampling = 2000,

adapt_delta = 0.95,

refresh = 0

)

\## Extracting the samples

MixedEffectsPKsamplefitdata<-MixedEffectsPKsamplefits$draws()

MixedEffectsPKsampledata<-as_draws_df(MixedEffectsPKsamplefitdata)

MixedEffectsPKposterior<-as.data.frame(MixedEffectsPKsampledata)

##To see MCMC summary

MixedEffectsPKHMCSummary<-MixedEffectsPKsamplefits$summary()

#Extract the median and the divergence diagnostics to showcase diagnostics

MixedEffectsPKHMCSummary<-as.data.frame(MixedEffectsPKHMCSummary[c(2:9),c(1,3,8,9,10)])

#Covariance matrix, standard error and relative standard error

#generate covariance matrix

covariancematrix<-cov(MixedEffectsPKposterior[c(2:9)])

#square root of the diagonal elements

SEvalues<-sqrt(diag(covariancematrix))

#obtain MXRSE values

RSEvalues<-(SEvalues/MixedEffectsPKHMCSummary$median)*100

#Begin to form summary table

MixedEffectsSummarydata<-cbind(MixedEffectsPKHMCSummary,SEvalues,RSEvalues)

#To highlight how to extract the standard error and 95%CDI the following commands can be used to extract a summary of the fixed effects and the constant error terms

##95CDI extraction and 95CDIRA calculation

\## Can be done manually but as some models may have many parameters better to create a function

interestedposterior<-MixedEffectsPKposterior[2:9]

credibleintervals<-function(interestedposterior){

intervallist<-c()

for(i in 1:ncol(interestedposterior)){

interval<-as.numeric(hdi(interestedposterior[,i]))

intervallist<-c(intervallist,interval)

}

return(cbind(intervallist[seq(1,length(intervallist),2)],intervallist[seq(2,length(intervallist),2)]))

}

cdi95list<-credibleintervals(interestedposterior)

MixedEffectsSummarydata<-cbind(MixedEffectsSummarydata,cdi95list)

\## generating the interval ratios

\# rationale: measure how far the MLE values are from the intervals, the closer the intervals are to the MLE, the shorter the interval - increased certainty. values >0.7 high precision, 0.5-0.7 moderate precision, <0.01, (to 3 decimal places) low precision, <0.001 very low precision

LBratios<-MixedEffectsSummarydata[,8]/MixedEffectsSummarydata[,2]

UBratios<-MixedEffectsSummarydata[,2]/MixedEffectsSummarydata[,9]

#calculate average ratio as well

AVGratios<-(LBratios+UBratios)/2

#bind to summary table

MixedEffectsSummarydata<-as.data.frame(cbind(MixedEffectsSummarydata,LBratios,UBratios,AVGratios))

#Column names for summary table

colnames(MixedEffectsSummarydata)<-c("Parameters","Median","Rhat","ESS_Bulk","ESS_Tail","SE","RSE","95_CDI_LB","95_CDI_UB", "95_CDI_Ratio_LB","95_CDI_Ratio_UB","95_CDI_Ratio_AVRG")

###End of script

Population SOE model

\### Commencement of script

library(rstan)

library(bayestestR)

library(posterior)

library(readxl)

library(dplyr)

library(tidyr)

library(ggplot2)

library(ggpubr)

library(HDInterval)

library(deSolve)

library(cmdstanr)

MixedEffectsSOE_model="

data {

int<lower=1> T;

int<lower=1> N_ID;

matrix[N_ID,T] y1;

array[T] real ts;

real A1; //initial estimates and SDs (1 is often used as the typical SD)

real B1;

real B2;

}

parameters {

real<lower=0> A1_POP;

// B1_POP and B2_POP declared jointly as positive_ordered to enforce

// 0 < B1_POP < B2_POP, breaking the label-switching symmetry between

// the two exponential rate constants. Aliases below in transformed parameters.

positive_ordered[2] B_POP;

real <lower=0> A1_OMEGA; //To make these distributions half-normal

real <lower=0> B1_OMEGA;

real <lower=0> B2_OMEGA;

real <lower=0> a; //

real <lower=0> b; //

vector[N_ID] A1_nv; // the eta values for estimating the individual parameter estimates

vector[N_ID] B1_nv;

vector[N_ID] B2_nv;

}

transformed parameters {

// Aliases so the rest of the model reads as before

real<lower=0> B1_POP = B_POP[1];

real<lower=0> B2_POP = B_POP[2];

vector <lower=0> [N_ID] A1_IND;

vector <lower=0>[N_ID] B1_IND;

vector <lower=0>[N_ID] B2_IND;

vector[N_ID] A1_ETA;

vector[N_ID] B1_ETA;

vector[N_ID] B2_ETA;

A1_ETA = A1_OMEGA * A1_nv;

B1_ETA = B1_OMEGA * B1_nv;

B2_ETA = B2_OMEGA * B2_nv;

for(i in 1:N_ID) {

A1_IND[i]=A1_POP*exp(A1_ETA[i]);

B1_IND[i]=B1_POP*exp(B1_ETA[i]);

B2_IND[i]=B2_POP*exp(B2_ETA[i]);

}

}

model {

A1_POP ~ lognormal(log(A1),0.5);

// Priors are applied to the ordered elements directly

B_POP[1] ~ lognormal(log(B1),0.5);

B_POP[2] ~ lognormal(log(B2),0.5);

A1_OMEGA~ normal(0,0.5);

B1_OMEGA~ normal(0,0.5);

B2_OMEGA ~ normal(0,0.5);

a ~ normal(0,1); // combined error model terms

b ~ normal(0,0.3);

for(i in 1:N_ID) {

A1_nv[i] ~ normal(0,1);

B1_nv[i] ~ normal(0,1);

B2_nv[i] ~ normal(0,1);

for (t in 1:T) {

// Use ts[t] (actual observation time, e.g. 0.5, 1, 2, ..., 24 hr), not the

// loop index t (1..11). Otherwise B1 and B2 absorb the time-axis rescaling.

y1[i,t] ~ normal(A1_IND[i]*exp(-B1_IND[i]*ts[t])+(-A1_IND[i])*exp(-B2_IND[i]*ts[t]),(a+b*(A1_IND[i]*exp(-B1_IND[i]*ts[t])+(-A1_IND[i])*exp(-B2_IND[i]*ts[t]))));

}

}

}

"

\## Converting the model into a cmdstan model

MixedEffectsSOEmodel<-write_stan_file(MixedEffectsSOE_model)

MixedEffectsSOEmodel<-cmdstan_model(MixedEffectsSOEmodel)

\## Data for the cmdstan model: everything that was declared in the data section needs to be included

MixedEffectsSOEdata<-list(T=length(unique(Time)),y1=as.matrix(t(pkdata)),ts=unique(Time),A1=1,

B1=1,B2=10,N_ID=50)# The parameters (KA,CL and V) are defined by the user, feel free to change them

\## Hamiltonian Monte Carlo sampling

##Note there may be some metropolis proposal rejections but these are quite rare,

MixedEffectsSOEsamplefits <- MixedEffectsSOEmodel$sample(

data = MixedEffectsSOEdata,

chains = 4,

parallel_chains = 4,

iter_warmup = 1500,

iter_sampling = 2000,

adapt_delta = 0.95,

refresh = 0

) # 4 chains in parallel; average wall time ~1 minute (no ODE solver).

\## Extracting the samples

MixedEffectsSOEsamplefitdata<-MixedEffectsSOEsamplefits$draws()

MixedEffectsSOEsampledata<-as_draws_df(MixedEffectsSOEsamplefitdata)

MixedEffectsSOEposterior<-as.data.frame(MixedEffectsSOEsampledata)

\#

MixedEffectsSOEHMCSummary<-MixedEffectsSOEsamplefits$summary()

MixedEffectsSOEHMCSummary<-as.data.frame(MixedEffectsSOEHMCSummary[c(2:9),c(1,3,8,9,10)])

#Covariance matrix, standard error and relative standard error

#generate covariance matrix

covariancematrix<-cov(MixedEffectsSOEposterior[c(2:9)])

#square root of the diagonal elements

SEvalues<-sqrt(diag(covariancematrix))

#obtain MXRSE values

RSEvalues<-(SEvalues/MixedEffectsSOEHMCSummary$median)*100

#Begin to form summary table

MixedEffectsSOESummarydata<-cbind(MixedEffectsSOEHMCSummary,SEvalues,RSEvalues)

cdi95list<-credibleintervals(MixedEffectsSOEposterior[2:9])

\## can add this to the PopSummarydata

MixedEffectsSOESummarydata<-cbind(MixedEffectsSOESummarydata,cdi95list)

\## generating the interval ratios

\# rationale: measure how far the MLE values are from the intervals, the B1oser the intervals are to the MLE, the shorter the interval - increased certainty. values >0.7 high precision, 0.5-0.7 moderate precision, <0.01, (to 3 decimal places) low precision, <0.001 very low precision

LBratios<-MixedEffectsSOESummarydata[,8]/MixedEffectsSOESummarydata[,2]

UBratios<-MixedEffectsSOESummarydata[,2]/MixedEffectsSOESummarydata[,9]

#calculate average ratio as well

AVGratios<-(LBratios+UBratios)/2

#bind to summary table

MixedEffectsSOESummarydata<-as.data.frame(cbind(MixedEffectsSOESummarydata,LBratios,UBratios,AVGratios))

#Column names for summary table

colnames(MixedEffectsSOESummarydata)<-c("Parameters","Median","Rhat","ESS_Bulk","ESS_Tail","SE","RSE","95_CDI_LB","95_CDI_UB", "95_CDI_Ratio_LB","95_CDI_Ratio_UB","95_CDI_Ratio_AVRG")

###End of script

Individual parameters and random effect estimates

| Parameters | Median | Rhat | ESS_Bulk | ESS_Tail | RSE | 95CDILB | 95CDIUB | 95CDIRALB | 95CDIRAUB |
| --- | --- | --- | --- | --- | --- | --- | --- | --- | --- |
| KA_IND[1] | 0.333572 | 1.000791 | 8513.535 | 5065.124 | 12.11486 | 0.262252 | 0.423002 | 0.786195 | 0.788581 |
| KA_IND[2] | 0.306646 | 1.002294 | 1229.188 | 2815.705 | 13.92886 | 0.213078 | 0.377492 | 0.694866 | 0.812323 |
| KA_IND[3] | 0.312315 | 1.002531 | 1492.236 | 2489.051 | 13.16053 | 0.222689 | 0.381531 | 0.713029 | 0.818582 |
| KA_IND[4] | 0.317629 | 1.002257 | 2413.348 | 3761.646 | 12.67457 | 0.232504 | 0.391117 | 0.731998 | 0.812109 |
| KA_IND[5] | 0.313813 | 1.000991 | 2474.857 | 3782.091 | 12.12065 | 0.23547 | 0.385374 | 0.750351 | 0.814306 |
| KA_IND[6] | 0.333498 | 1.001375 | 8691.859 | 5406.732 | 11.85898 | 0.264752 | 0.420386 | 0.793864 | 0.793312 |
| KA_IND[7] | 0.314357 | 1.001996 | 1735.956 | 3164.338 | 12.72475 | 0.229311 | 0.385443 | 0.72946 | 0.815572 |
| KA_IND[8] | 0.299849 | 1.003969 | 904.7652 | 1715.997 | 16.09867 | 0.192474 | 0.37157 | 0.641903 | 0.806979 |
| KA_IND[9] | 0.33 | 1.000538 | 7205.999 | 5564.85 | 11.72895 | 0.258566 | 0.412327 | 0.783534 | 0.800336 |
| KA_IND[10] | 0.345033 | 1.001963 | 3230.656 | 5002.863 | 12.68329 | 0.272381 | 0.441475 | 0.789434 | 0.781544 |
| KA_IND[11] | 0.316421 | 1.001488 | 2273.633 | 3301.144 | 12.33694 | 0.229556 | 0.382705 | 0.725478 | 0.826801 |
| KA_IND[12] | 0.320285 | 1.00099 | 3375.427 | 3998.302 | 11.73054 | 0.240581 | 0.390133 | 0.751146 | 0.820963 |
| KA_IND[13] | 0.341633 | 1.001332 | 7129.627 | 5687.426 | 12.78834 | 0.273321 | 0.444536 | 0.800045 | 0.768516 |
| KA_IND[14] | 0.319975 | 1.001509 | 3657.077 | 3145.465 | 11.96122 | 0.236398 | 0.392377 | 0.738799 | 0.81548 |
| KA_IND[15] | 0.306739 | 1.002275 | 1589.051 | 3305.974 | 12.82577 | 0.224897 | 0.375755 | 0.733187 | 0.816326 |
| KA_IND[16] | 0.344624 | 1.002199 | 1954.627 | 2397.234 | 13.2291 | 0.272876 | 0.44641 | 0.791807 | 0.771988 |
| KA_IND[17] | 0.320555 | 1.002158 | 3535.001 | 4304.269 | 12.13003 | 0.235138 | 0.391231 | 0.733534 | 0.81935 |
| KA_IND[18] | 0.342232 | 1.001333 | 5306.002 | 5589.773 | 12.59102 | 0.273279 | 0.439216 | 0.79852 | 0.779188 |
| KA_IND[19] | 0.352905 | 1.004111 | 1438.198 | 2724.695 | 14.79796 | 0.282594 | 0.477473 | 0.800764 | 0.739111 |
| KA_IND[20] | 0.315479 | 1.001595 | 2360.506 | 3217.614 | 12.1482 | 0.232878 | 0.386033 | 0.738174 | 0.817232 |
| KA_IND[21] | 0.31344 | 1.00221 | 1870.878 | 3453.573 | 12.94687 | 0.22397 | 0.383251 | 0.714554 | 0.817844 |
| KA_IND[22] | 0.305805 | 1.003732 | 1280.545 | 2281.782 | 13.70176 | 0.217076 | 0.375613 | 0.709852 | 0.814147 |
| KA_IND[23] | 0.308015 | 1.002959 | 1312.704 | 2715.05 | 13.30995 | 0.22091 | 0.379049 | 0.717207 | 0.8126 |
| KA_IND[24] | 0.339391 | 1.001643 | 5453.389 | 4572.301 | 12.44615 | 0.269761 | 0.433294 | 0.794838 | 0.78328 |
| KA_IND[25] | 0.331023 | 1.000995 | 7714.426 | 5092.82 | 11.66179 | 0.258004 | 0.41097 | 0.779416 | 0.805467 |
| KA_IND[26] | 0.335937 | 1.001548 | 8277.558 | 5674.078 | 12.06191 | 0.262468 | 0.423695 | 0.781299 | 0.792876 |
| KA_IND[27] | 0.314103 | 1.001042 | 2316.737 | 4405.589 | 12.30208 | 0.235394 | 0.387726 | 0.749416 | 0.810115 |
| KA_IND[28] | 0.329401 | 1.001846 | 9458.699 | 4787.818 | 11.54139 | 0.259972 | 0.409356 | 0.789226 | 0.804682 |
| KA_IND[29] | 0.299958 | 1.003392 | 956.4993 | 1917.791 | 15.8756 | 0.197328 | 0.37406 | 0.657852 | 0.801897 |
| KA_IND[30] | 0.335044 | 1.001277 | 8769.879 | 6119.071 | 11.95368 | 0.263976 | 0.424996 | 0.787885 | 0.788345 |
| KA_IND[31] | 0.31327 | 1.001232 | 2033.94 | 3181.279 | 12.37335 | 0.230074 | 0.379965 | 0.734428 | 0.82447 |
| KA_IND[32] | 0.351938 | 1.003852 | 1759.904 | 3691.534 | 13.95492 | 0.279412 | 0.46361 | 0.793926 | 0.759125 |
| KA_IND[33] | 0.364468 | 1.002909 | 1299.671 | 3628.82 | 16.10853 | 0.282255 | 0.497376 | 0.774431 | 0.732781 |
| KA_IND[34] | 0.314155 | 1.001826 | 2074.607 | 3678.973 | 12.01127 | 0.231338 | 0.380551 | 0.736381 | 0.825527 |
| KA_IND[35] | 0.323692 | 1.00172 | 8866.984 | 5381.743 | 11.41912 | 0.24905 | 0.397462 | 0.769406 | 0.814396 |
| KA_IND[36] | 0.311661 | 1.002319 | 1600.693 | 2822.879 | 13.37574 | 0.22281 | 0.386908 | 0.71491 | 0.805519 |
| KA_IND[37] | 0.291964 | 1.006346 | 813.1857 | 1790.311 | 17.22732 | 0.181524 | 0.36464 | 0.621734 | 0.800691 |
| KA_IND[38] | 0.344258 | 1.002235 | 3450.677 | 4305.422 | 12.87054 | 0.278957 | 0.450229 | 0.810313 | 0.764629 |
| KA_IND[39] | 0.326694 | 1.001273 | 6096.834 | 4213.14 | 11.73582 | 0.251259 | 0.404209 | 0.769095 | 0.80823 |
| KA_IND[40] | 0.336381 | 1.001574 | 5972.411 | 4478.048 | 11.70022 | 0.261963 | 0.418234 | 0.778767 | 0.80429 |
| KA_IND[41] | 0.331088 | 1.000506 | 7353.991 | 4683.447 | 11.88816 | 0.251895 | 0.409247 | 0.760809 | 0.809018 |
| KA_IND[42] | 0.339765 | 1.001696 | 5308.772 | 5028.964 | 12.30944 | 0.269101 | 0.433786 | 0.79202 | 0.783256 |
| KA_IND[43] | 0.327948 | 1.001139 | 4457.924 | 3982.406 | 12.44354 | 0.249678 | 0.414112 | 0.761334 | 0.791932 |
| KA_IND[44] | 0.333636 | 1.001279 | 9693.131 | 5203.202 | 12.11789 | 0.260014 | 0.419711 | 0.779334 | 0.794919 |
| KA_IND[45] | 0.309499 | 1.00261 | 1346.842 | 2414.103 | 14.35915 | 0.211472 | 0.383564 | 0.683273 | 0.806905 |
| KA_IND[46] | 0.308156 | 1.003425 | 1264.795 | 2928.685 | 13.16573 | 0.219975 | 0.37586 | 0.713842 | 0.819869 |
| KA_IND[47] | 0.306712 | 1.002374 | 1271.401 | 2555.477 | 13.67191 | 0.212952 | 0.373224 | 0.694305 | 0.82179 |
| KA_IND[48] | 0.323262 | 1.00045 | 5127.756 | 4110.046 | 12.03398 | 0.241917 | 0.396273 | 0.748361 | 0.815757 |
| KA_IND[49] | 0.308504 | 1.00287 | 1271.63 | 2315.168 | 13.87318 | 0.214435 | 0.378696 | 0.695081 | 0.814647 |
| KA_IND[50] | 0.314144 | 1.001824 | 2133.498 | 3408.162 | 12.14004 | 0.231916 | 0.382234 | 0.738247 | 0.821865 |
| CL_IND[1] | 0.852 | 1.00018 | 12935.98 | 5886.218 | 7.975671 | 0.719119 | 0.986456 | 0.844036 | 0.863698 |
| CL_IND[2] | 1.111047 | 1.000335 | 10224.24 | 6223.297 | 8.274619 | 0.933824 | 1.297271 | 0.84049 | 0.856449 |
| CL_IND[3] | 1.207528 | 1.000863 | 12801.55 | 4821.9 | 8.344001 | 1.013296 | 1.408278 | 0.839149 | 0.85745 |
| CL_IND[4] | 1.201353 | 1.000188 | 13303.85 | 6506.923 | 8.163669 | 1.01276 | 1.396452 | 0.843017 | 0.860289 |
| CL_IND[5] | 0.91978 | 1.00132 | 13628.01 | 5854.408 | 8.16414 | 0.77097 | 1.067861 | 0.838212 | 0.861329 |
| CL_IND[6] | 0.877775 | 1.000353 | 12585.04 | 5711.857 | 7.433511 | 0.754326 | 1.008422 | 0.859362 | 0.870443 |
| CL_IND[7] | 1.080073 | 1.000427 | 12043.78 | 6468.475 | 7.933538 | 0.91568 | 1.247751 | 0.847795 | 0.865616 |
| CL_IND[8] | 1.719277 | 1.000781 | 10778.19 | 5830.669 | 9.401184 | 1.39487 | 2.023521 | 0.811311 | 0.849646 |
| CL_IND[9] | 0.76797 | 1.000643 | 12534.2 | 5955.102 | 7.597726 | 0.65798 | 0.885882 | 0.856779 | 0.866899 |
| CL_IND[10] | 0.825301 | 1.000682 | 12797.02 | 5588.687 | 7.404253 | 0.706931 | 0.945245 | 0.856573 | 0.873109 |
| CL_IND[11] | 1.112323 | 1.000023 | 12111.49 | 5856.176 | 8.038713 | 0.938928 | 1.2867 | 0.844115 | 0.864477 |
| CL_IND[12] | 0.927295 | 1.000208 | 12315.08 | 5537.175 | 7.851669 | 0.784488 | 1.070773 | 0.845997 | 0.866005 |
| CL_IND[13] | 0.908309 | 1.00095 | 11971.12 | 5992.365 | 7.970112 | 0.779474 | 1.059321 | 0.85816 | 0.857445 |
| CL_IND[14] | 0.97157 | 0.999847 | 13864.2 | 6232.112 | 7.703631 | 0.818808 | 1.1132 | 0.842768 | 0.872772 |
| CL_IND[15] | 0.977293 | 1.000303 | 11839.91 | 5334.111 | 8.104402 | 0.824751 | 1.135365 | 0.843913 | 0.860775 |
| CL_IND[16] | 0.621541 | 1.000596 | 10953.69 | 6503.533 | 7.381459 | 0.529995 | 0.709294 | 0.852712 | 0.87628 |
| CL_IND[17] | 1.077875 | 1.001426 | 12342.38 | 5671.922 | 7.920632 | 0.918608 | 1.252334 | 0.852239 | 0.860693 |
| CL_IND[18] | 1.05691 | 1.001175 | 13960.81 | 5455.676 | 7.603696 | 0.894749 | 1.214972 | 0.846571 | 0.869905 |
| CL_IND[19] | 0.645966 | 1.000249 | 11664.64 | 5227.757 | 7.318242 | 0.551695 | 0.737703 | 0.854062 | 0.875646 |
| CL_IND[20] | 1.101844 | 1.000844 | 12052.34 | 5409.68 | 8.170469 | 0.92293 | 1.277819 | 0.837623 | 0.862285 |
| CL_IND[21] | 1.05688 | 0.999942 | 13187.2 | 5989.62 | 8.040029 | 0.894636 | 1.226513 | 0.846488 | 0.861694 |
| CL_IND[22] | 1.312872 | 1.000071 | 12374.28 | 6085.018 | 8.352654 | 1.102872 | 1.53102 | 0.840045 | 0.857514 |
| CL_IND[23] | 0.913068 | 1.000354 | 12304.34 | 5632.563 | 8.501287 | 0.768693 | 1.073912 | 0.841879 | 0.850226 |
| CL_IND[24] | 1.236907 | 1.000171 | 13495.97 | 6337.882 | 7.698473 | 1.060896 | 1.433983 | 0.8577 | 0.862568 |
| CL_IND[25] | 0.996521 | 1.001201 | 12895.69 | 5898.928 | 7.743413 | 0.847527 | 1.150151 | 0.850486 | 0.866426 |
| CL_IND[26] | 0.902173 | 1.000038 | 12301.73 | 6272.669 | 7.424996 | 0.76935 | 1.032581 | 0.852775 | 0.873706 |
| CL_IND[27] | 1.50588 | 0.999918 | 11835.59 | 5396.619 | 8.313526 | 1.261531 | 1.752957 | 0.837737 | 0.859051 |
| CL_IND[28] | 0.877273 | 1.001357 | 13502.16 | 6166.703 | 7.555357 | 0.748511 | 1.009245 | 0.853225 | 0.869237 |
| CL_IND[29] | 1.387962 | 1.000154 | 11118.78 | 5907.329 | 8.775891 | 1.16051 | 1.638728 | 0.836125 | 0.846975 |
| CL_IND[30] | 1.073069 | 0.999882 | 12089.24 | 4588.862 | 7.625146 | 0.910798 | 1.237804 | 0.848779 | 0.866913 |
| CL_IND[31] | 1.179223 | 1.000201 | 12369.21 | 6008.655 | 8.006267 | 0.992207 | 1.362898 | 0.841408 | 0.865232 |
| CL_IND[32] | 0.884492 | 1.000798 | 12817.05 | 5643.184 | 7.175492 | 0.755456 | 1.004276 | 0.854113 | 0.880726 |
| CL_IND[33] | 0.869448 | 1.003738 | 12833.1 | 5341.436 | 7.088308 | 0.749662 | 0.9876 | 0.862227 | 0.880365 |
| CL_IND[34] | 1.156024 | 1.000523 | 12170.25 | 5883.408 | 7.93267 | 0.977747 | 1.334232 | 0.845784 | 0.866434 |
| CL_IND[35] | 0.879454 | 1.000999 | 14496.9 | 5662.874 | 7.87769 | 0.742653 | 1.014244 | 0.844447 | 0.867103 |
| CL_IND[36] | 1.444003 | 1.000601 | 13322.31 | 5959.251 | 8.387212 | 1.212721 | 1.685845 | 0.839833 | 0.856546 |
| CL_IND[37] | 1.770941 | 1.000173 | 9357.598 | 6305.324 | 9.476161 | 1.461666 | 2.126632 | 0.825362 | 0.832744 |
| CL_IND[38] | 0.97561 | 0.999883 | 13180 | 5183.212 | 7.177817 | 0.838896 | 1.1158 | 0.859868 | 0.874359 |
| CL_IND[39] | 1.011909 | 1.000783 | 13945.86 | 5797.943 | 7.693284 | 0.863424 | 1.163398 | 0.853263 | 0.869787 |
| CL_IND[40] | 0.930877 | 0.999905 | 12413.32 | 5722.704 | 7.254043 | 0.790479 | 1.054429 | 0.849176 | 0.882826 |
| CL_IND[41] | 1.099696 | 0.999827 | 13497.07 | 6330.978 | 7.74066 | 0.942493 | 1.277118 | 0.857049 | 0.861076 |
| CL_IND[42] | 1.230645 | 1.000136 | 12289.08 | 5682.784 | 7.586498 | 1.049726 | 1.414603 | 0.852988 | 0.869958 |
| CL_IND[43] | 1.236767 | 0.99986 | 12679 | 5939.17 | 8.10907 | 1.048954 | 1.441173 | 0.848142 | 0.858167 |
| CL_IND[44] | 0.829454 | 0.999853 | 12313.86 | 5900.113 | 7.90353 | 0.700844 | 0.958307 | 0.844946 | 0.865541 |
| CL_IND[45] | 1.369992 | 0.999994 | 12133.06 | 5752.922 | 8.56564 | 1.156393 | 1.622655 | 0.844087 | 0.844291 |
| CL_IND[46] | 1.092966 | 1.000301 | 10992.58 | 5636.941 | 8.210185 | 0.912065 | 1.264935 | 0.834486 | 0.864049 |
| CL_IND[47] | 0.920701 | 0.999875 | 11771.87 | 5337.283 | 8.434197 | 0.763214 | 1.067257 | 0.828948 | 0.86268 |
| CL_IND[48] | 1.583538 | 1.000432 | 12566.86 | 6384.908 | 8.489918 | 1.319722 | 1.845149 | 0.833401 | 0.858217 |
| CL_IND[49] | 1.442365 | 1.001765 | 12142.88 | 6059.482 | 8.438148 | 1.210522 | 1.690715 | 0.839262 | 0.853109 |
| CL_IND[50] | 0.915829 | 1.001203 | 11861.54 | 5756.64 | 7.926312 | 0.779021 | 1.063696 | 0.850618 | 0.860987 |
| V_IND[1] | 7.844061 | 1.000339 | 8751.852 | 6252.648 | 9.806386 | 6.398042 | 9.405544 | 0.815654 | 0.833983 |
| V_IND[2] | 9.084986 | 1.002805 | 1317.654 | 3191.972 | 12.79353 | 7.076334 | 11.49212 | 0.778904 | 0.790541 |
| V_IND[3] | 8.870529 | 1.001897 | 1613.211 | 3260.972 | 12.18665 | 6.937703 | 11.05893 | 0.782107 | 0.802115 |
| V_IND[4] | 8.624249 | 1.000781 | 2289.617 | 4577.824 | 11.55344 | 6.839826 | 10.64238 | 0.793092 | 0.810369 |
| V_IND[5] | 8.148533 | 1.000742 | 3530.14 | 6370.944 | 10.30502 | 6.557058 | 9.804476 | 0.804692 | 0.831103 |
| V_IND[6] | 7.382082 | 1.000272 | 8837.215 | 5834.092 | 9.570704 | 6.051682 | 8.829315 | 0.81978 | 0.836088 |
| V_IND[7] | 8.581066 | 1.001173 | 2095.223 | 4031.825 | 11.34496 | 6.877202 | 10.63064 | 0.801439 | 0.807202 |
| V_IND[8] | 9.831977 | 1.004257 | 951.3898 | 1715.268 | 16.12181 | 6.970369 | 12.92836 | 0.708949 | 0.760497 |
| V_IND[9] | 6.89973 | 1.000326 | 4367.04 | 5311.431 | 9.968978 | 5.604129 | 8.285046 | 0.812224 | 0.832793 |
| V_IND[10] | 7.139597 | 1.001211 | 3988.43 | 6112.546 | 9.750629 | 5.802843 | 8.524523 | 0.812769 | 0.837536 |
| V_IND[11] | 8.439919 | 1.001607 | 2514.019 | 5757.247 | 11.14809 | 6.775979 | 10.33236 | 0.802849 | 0.816843 |
| V_IND[12] | 8.084488 | 1.000353 | 4147.245 | 5213.952 | 10.24063 | 6.584277 | 9.826691 | 0.814433 | 0.822707 |
| V_IND[13] | 7.926966 | 1.000537 | 7726.559 | 6403.33 | 9.934952 | 6.475236 | 9.563842 | 0.816862 | 0.828847 |
| V_IND[14] | 8.128534 | 1.000039 | 3901.672 | 6216.917 | 10.2605 | 6.558048 | 9.812491 | 0.806793 | 0.828386 |
| V_IND[15] | 8.309751 | 1.000882 | 2482.557 | 5209.02 | 10.89663 | 6.736878 | 10.19993 | 0.81072 | 0.814687 |
| V_IND[16] | 6.252174 | 1.001708 | 1632.54 | 1718.39 | 10.9216 | 5.034289 | 7.702736 | 0.805206 | 0.811682 |
| V_IND[17] | 8.344437 | 1.000638 | 3224.155 | 4766.085 | 10.8518 | 6.753489 | 10.25532 | 0.80934 | 0.813669 |
| V_IND[18] | 7.395718 | 1.000598 | 6860.605 | 5737.265 | 10.25336 | 5.942285 | 8.900681 | 0.803476 | 0.830916 |
| V_IND[19] | 6.369212 | 1.002039 | 1416.089 | 2178.801 | 10.91884 | 5.199135 | 7.867643 | 0.816292 | 0.809545 |
| V_IND[20] | 8.250268 | 1.000486 | 3168.468 | 4496.065 | 10.91745 | 6.652856 | 10.14331 | 0.806381 | 0.813371 |
| V_IND[21] | 8.592457 | 1.00138 | 2196.256 | 4841.386 | 11.56205 | 6.727495 | 10.54967 | 0.782954 | 0.814477 |
| V_IND[22] | 8.825871 | 1.00206 | 1538.835 | 3696.424 | 12.65484 | 6.843201 | 11.06578 | 0.775357 | 0.797582 |
| V_IND[23] | 8.882732 | 1.00242 | 1476.241 | 3231.031 | 11.73487 | 6.903394 | 10.88455 | 0.77717 | 0.816086 |
| V_IND[24] | 7.292761 | 1.000208 | 5908.759 | 6358.348 | 10.15797 | 5.836356 | 8.684058 | 0.800294 | 0.839787 |
| V_IND[25] | 7.812854 | 1.000722 | 7672.624 | 6496.76 | 9.694275 | 6.424725 | 9.403649 | 0.822327 | 0.830832 |
| V_IND[26] | 7.547772 | 1.000177 | 8834.177 | 6669.428 | 9.72062 | 6.089098 | 8.983674 | 0.806741 | 0.840165 |
| V_IND[27] | 8.310134 | 1.000912 | 2575.664 | 5597.708 | 11.69505 | 6.542348 | 10.29575 | 0.787274 | 0.807142 |
| V_IND[28] | 7.497209 | 1.000491 | 8874.537 | 6058.93 | 9.604238 | 6.045205 | 8.886749 | 0.806327 | 0.843639 |
| V_IND[29] | 9.728886 | 1.003173 | 978.7445 | 2106.153 | 15.04761 | 6.970745 | 12.47874 | 0.7165 | 0.779637 |
| V_IND[30] | 7.540935 | 1.000123 | 8588.356 | 5988.308 | 10.16464 | 6.100631 | 9.096776 | 0.809002 | 0.828968 |
| V_IND[31] | 8.464666 | 1.000623 | 2274.242 | 5755.181 | 11.28886 | 6.812273 | 10.44661 | 0.804789 | 0.810279 |
| V_IND[32] | 6.673685 | 1.002478 | 1852.788 | 4469.093 | 10.57198 | 5.35835 | 8.077969 | 0.802907 | 0.826159 |
| V_IND[33] | 6.764476 | 1.002153 | 1703.296 | 4507.179 | 11.04984 | 5.320189 | 8.183665 | 0.786489 | 0.826583 |
| V_IND[34] | 8.236274 | 1.000546 | 2341.654 | 5187.538 | 10.86899 | 6.588345 | 9.993885 | 0.799918 | 0.824131 |
| V_IND[35] | 7.652239 | 1.000823 | 9794.319 | 6476.274 | 9.677295 | 6.293725 | 9.159612 | 0.822468 | 0.835433 |
| V_IND[36] | 8.85563 | 1.001991 | 1616.736 | 3780.977 | 12.78205 | 6.85704 | 11.16377 | 0.774314 | 0.793247 |
| V_IND[37] | 10.15746 | 1.005467 | 886.7294 | 1887.001 | 16.91123 | 7.03632 | 13.37712 | 0.692725 | 0.759316 |
| V_IND[38] | 6.999191 | 1.000781 | 3608.142 | 5230.593 | 10.1679 | 5.630385 | 8.350621 | 0.804434 | 0.838164 |
| V_IND[39] | 7.951187 | 1.000542 | 6327.837 | 5741.883 | 10.05316 | 6.488354 | 9.577181 | 0.816023 | 0.830222 |
| V_IND[40] | 6.963698 | 1.000843 | 4400.834 | 6485.992 | 9.998624 | 5.637957 | 8.320625 | 0.809621 | 0.83692 |
| V_IND[41] | 7.886765 | 1.000233 | 7095.416 | 5597.277 | 10.30957 | 6.354814 | 9.534969 | 0.805757 | 0.827141 |
| V_IND[42] | 7.405183 | 1.000465 | 7107.014 | 5539.885 | 10.40951 | 5.877069 | 8.914887 | 0.793643 | 0.830654 |
| V_IND[43] | 8.39051 | 1.000476 | 3537.866 | 4827.959 | 11.36247 | 6.693944 | 10.36568 | 0.797799 | 0.809451 |
| V_IND[44] | 7.843695 | 1.00061 | 8799.218 | 6402.148 | 9.547252 | 6.439963 | 9.380693 | 0.821037 | 0.836153 |
| V_IND[45] | 9.332388 | 1.002317 | 1252.992 | 2988.684 | 13.48588 | 6.923441 | 11.70171 | 0.741872 | 0.797524 |
| V_IND[46] | 8.792873 | 1.001866 | 1666.659 | 3621.039 | 11.88114 | 6.975417 | 10.93733 | 0.793303 | 0.803932 |
| V_IND[47] | 8.830021 | 1.001741 | 1420.077 | 3184.626 | 11.69694 | 7.05038 | 10.99426 | 0.798456 | 0.803148 |
| V_IND[48] | 8.213368 | 1.000686 | 4249.757 | 6214.141 | 11.54019 | 6.493722 | 10.15361 | 0.790628 | 0.808911 |
| V_IND[49] | 9.14596 | 1.002122 | 1348.04 | 2981.78 | 13.34196 | 6.90084 | 11.54503 | 0.754523 | 0.792199 |
| V_IND[50] | 8.247059 | 1.00134 | 2903.722 | 5684.304 | 10.37477 | 6.693026 | 9.998827 | 0.811565 | 0.824803 |
| KA_ETA[1] | 0.019112 | 1.002137 | 5473.054 | 5222.612 | 552.607 | -0.1562 | 0.275408 | -8.17293 | 0.069394 |
| KA_ETA[2] | -0.04551 | 1.001811 | 1580.053 | 3337.757 | -273.007 | -0.35695 | 0.129764 | 7.843537 | -0.3507 |
| KA_ETA[3] | -0.02902 | 1.002952 | 2105.875 | 3305.124 | -389.512 | -0.30428 | 0.148902 | 10.48487 | -0.1949 |
| KA_ETA[4] | -0.01434 | 1.003172 | 4714.515 | 4677.984 | -755.593 | -0.26756 | 0.185868 | 18.65833 | -0.07715 |
| KA_ETA[5] | -0.02283 | 1.002042 | 5463.906 | 4829.575 | -454.461 | -0.27435 | 0.152128 | 12.01683 | -0.15008 |
| KA_ETA[6] | 0.019467 | 1.001621 | 3674.308 | 4050.613 | 547.9147 | -0.16977 | 0.261424 | -8.72087 | 0.074467 |
| KA_ETA[7] | -0.02333 | 1.00155 | 2789.429 | 3779.513 | -468.875 | -0.27844 | 0.17014 | 11.9362 | -0.13711 |
| KA_ETA[8] | -0.07114 | 1.003593 | 1018.83 | 2298.878 | -210.243 | -0.44406 | 0.110489 | 6.241842 | -0.64389 |
| KA_ETA[9] | 0.009989 | 1.002483 | 2648.97 | 3395.251 | 1073.411 | -0.1852 | 0.260406 | -18.5399 | 0.03836 |
| KA_ETA[10] | 0.052156 | 1.003324 | 1518.269 | 3695.668 | 230.8629 | -0.11916 | 0.343701 | -2.28475 | 0.151749 |
| KA_ETA[11] | -0.01686 | 1.002596 | 4535.183 | 4866.403 | -624.584 | -0.26612 | 0.173686 | 15.78288 | -0.09708 |
| KA_ETA[12] | -0.00782 | 1.004106 | 8565.697 | 5078.788 | -1292.89 | -0.23215 | 0.194118 | 29.66781 | -0.04031 |
| KA_ETA[13] | 0.043816 | 1.001423 | 3145.96 | 4075.952 | 259.4157 | -0.11901 | 0.327452 | -2.71607 | 0.133809 |
| KA_ETA[14] | -0.00895 | 1.004234 | 8222.342 | 5208.893 | -1145.98 | -0.2398 | 0.193962 | 26.79018 | -0.04615 |
| KA_ETA[15] | -0.04577 | 1.001623 | 2491.83 | 4423.265 | -244.049 | -0.32363 | 0.119511 | 7.071252 | -0.38295 |
| KA_ETA[16] | 0.048552 | 1.003981 | 1137.178 | 1967.692 | 261.8505 | -0.13349 | 0.36216 | -2.74939 | 0.134064 |
| KA_ETA[17] | -0.00619 | 1.004687 | 7371.497 | 5014.228 | -1675.13 | -0.24169 | 0.198192 | 39.06755 | -0.03121 |
| KA_ETA[18] | 0.044109 | 1.001889 | 2269.049 | 3776.606 | 260.4895 | -0.14371 | 0.307755 | -3.25815 | 0.143326 |
| KA_ETA[19] | 0.072567 | 1.005618 | 958.2867 | 1940.937 | 195.8139 | -0.09209 | 0.435485 | -1.26907 | 0.166635 |
| KA_ETA[20] | -0.01929 | 1.001501 | 5260.05 | 4457.571 | -541.783 | -0.26665 | 0.167929 | 13.82426 | -0.11486 |
| KA_ETA[21] | -0.02486 | 1.003155 | 2988.136 | 4346.849 | -445.847 | -0.30921 | 0.144553 | 12.43954 | -0.17196 |
| KA_ETA[22] | -0.04991 | 1.003295 | 1744.934 | 3703.664 | -241.569 | -0.35489 | 0.116154 | 7.110414 | -0.4297 |
| KA_ETA[23] | -0.04338 | 1.002356 | 1868.06 | 3793.55 | -269.198 | -0.32673 | 0.141297 | 7.531304 | -0.30703 |
| KA_ETA[24] | 0.035437 | 1.00371 | 2056.986 | 3421.253 | 319.5155 | -0.14262 | 0.306249 | -4.02457 | 0.115712 |
| KA_ETA[25] | 0.012622 | 1.003244 | 5422.208 | 4108.824 | 818.5424 | -0.17952 | 0.252756 | -14.2227 | 0.049937 |
| KA_ETA[26] | 0.027678 | 1.002451 | 3384.7 | 4224.241 | 393.8232 | -0.15706 | 0.282088 | -5.6747 | 0.098117 |
| KA_ETA[27] | -0.02272 | 1.000709 | 4588.837 | 5733.681 | -461.12 | -0.27268 | 0.16179 | 12.00095 | -0.14044 |
| KA_ETA[28] | 0.00926 | 1.00332 | 4922.21 | 4434.643 | 1104.16 | -0.17085 | 0.252647 | -18.4504 | 0.036652 |
| KA_ETA[29] | -0.06857 | 1.002895 | 1115.766 | 2427.976 | -213.428 | -0.42563 | 0.126494 | 6.207296 | -0.54208 |
| KA_ETA[30] | 0.02207 | 1.001388 | 3978.145 | 4273.198 | 490.7534 | -0.15628 | 0.284684 | -7.08128 | 0.077524 |
| KA_ETA[31] | -0.02593 | 1.001721 | 3809.753 | 4567.222 | -410.829 | -0.28713 | 0.140806 | 11.07499 | -0.18412 |
| KA_ETA[32] | 0.072233 | 1.00559 | 1052.854 | 2472.24 | 184.0634 | -0.09254 | 0.39844 | -1.28115 | 0.181289 |
| KA_ETA[33] | 0.110549 | 1.004659 | 904.8764 | 2574.459 | 138.6199 | -0.07868 | 0.461945 | -0.71173 | 0.239313 |
| KA_ETA[34] | -0.02109 | 1.001531 | 4298.143 | 4880.8 | -491.048 | -0.26779 | 0.160585 | 12.69487 | -0.13136 |
| KA_ETA[35] | -0.00105 | 1.002988 | 9561.003 | 4616.417 | -9635 | -0.21151 | 0.218271 | 201.3947 | -0.00481 |
| KA_ETA[36] | -0.02919 | 1.002903 | 2562.346 | 3706.948 | -396.84 | -0.30943 | 0.167133 | 10.5994 | -0.17467 |
| KA_ETA[37] | -0.09985 | 1.006354 | 906.812 | 2099.141 | -163.782 | -0.50812 | 0.081582 | 5.08879 | -1.22394 |
| KA_ETA[38] | 0.049254 | 1.002922 | 1517.95 | 3281.106 | 246.2518 | -0.11673 | 0.355967 | -2.36991 | 0.138365 |
| KA_ETA[39] | 0.004447 | 1.001946 | 8703.199 | 5104.879 | 2315.856 | -0.20041 | 0.228374 | -45.0695 | 0.019471 |
| KA_ETA[40] | 0.024031 | 1.004154 | 2196.773 | 3575.907 | 454.9477 | -0.14517 | 0.293963 | -6.04101 | 0.081747 |
| KA_ETA[41] | 0.012661 | 1.002073 | 6169.144 | 4627.429 | 819.1601 | -0.18529 | 0.242467 | -14.634 | 0.052219 |
| KA_ETA[42] | 0.038063 | 1.002537 | 2104.262 | 3258.165 | 293.4447 | -0.13097 | 0.309651 | -3.44092 | 0.122922 |
| KA_ETA[43] | 0.006816 | 1.002808 | 7943.169 | 4168.977 | 1551.666 | -0.20788 | 0.231053 | -30.498 | 0.0295 |
| KA_ETA[44] | 0.020003 | 1.002647 | 4977.065 | 4451.165 | 532.096 | -0.1695 | 0.271992 | -8.47388 | 0.073541 |
| KA_ETA[45] | -0.03696 | 1.002675 | 1800.677 | 2983.842 | -343.637 | -0.34149 | 0.161439 | 9.239161 | -0.22895 |
| KA_ETA[46] | -0.04089 | 1.002643 | 1883.521 | 4178.933 | -281.629 | -0.32682 | 0.132249 | 7.993196 | -0.30917 |
| KA_ETA[47] | -0.0455 | 1.002326 | 1738.826 | 3628.289 | -260.003 | -0.33016 | 0.137181 | 7.255832 | -0.3317 |
| KA_ETA[48] | -0.00113 | 1.004163 | 8920.995 | 5302.583 | -9131.84 | -0.21959 | 0.205998 | 194.355 | -0.00548 |
| KA_ETA[49] | -0.03931 | 1.003388 | 1725.977 | 2524.564 | -308.944 | -0.32742 | 0.152323 | 8.328258 | -0.2581 |
| KA_ETA[50] | -0.02221 | 1.003513 | 4314.616 | 4342.482 | -477.359 | -0.2804 | 0.156773 | 12.62274 | -0.14169 |
| CL_ETA[1] | -0.20949 | 1.000607 | 5385.106 | 4886.253 | -41.2814 | -0.37933 | -0.04198 | 1.810785 | 4.990007 |
| CL_ETA[2] | 0.054897 | 1.000438 | 5183.348 | 5822.797 | 163.4075 | -0.13415 | 0.215739 | -2.44359 | 0.254459 |
| CL_ETA[3] | 0.138043 | 1.000643 | 5167.193 | 4371.121 | 64.99079 | -0.03406 | 0.316051 | -0.2467 | 0.436776 |
| CL_ETA[4] | 0.132486 | 0.999956 | 5384.924 | 5256.17 | 66.88307 | -0.04761 | 0.298978 | -0.35938 | 0.44313 |
| CL_ETA[5] | -0.13194 | 1.001069 | 5575.159 | 5664.206 | -67.1336 | -0.31454 | 0.033709 | 2.383898 | -3.9142 |
| CL_ETA[6] | -0.18102 | 1.000641 | 4725.702 | 5374.169 | -45.1611 | -0.34256 | -0.02343 | 1.892351 | 7.726839 |
| CL_ETA[7] | 0.026563 | 1.000619 | 5669.451 | 6188.829 | 322.5106 | -0.14899 | 0.184456 | -5.60896 | 0.144006 |
| CL_ETA[8] | 0.492696 | 1.000689 | 5984.658 | 5170.061 | 20.12729 | 0.294742 | 0.678474 | 0.598223 | 0.726182 |
| CL_ETA[9] | -0.31524 | 1.000056 | 4861.291 | 5686.679 | -26.3593 | -0.47885 | -0.15478 | 1.518973 | 2.036721 |
| CL_ETA[10] | -0.24089 | 1.000454 | 4715.544 | 5577.332 | -34.0293 | -0.40263 | -0.0853 | 1.671388 | 2.823946 |
| CL_ETA[11] | 0.057139 | 1.000329 | 5160.764 | 5065.563 | 152.2057 | -0.11559 | 0.22308 | -2.02298 | 0.256137 |
| CL_ETA[12] | -0.12527 | 1.000564 | 5248.79 | 5577.626 | -67.633 | -0.29937 | 0.035877 | 2.389908 | -3.49149 |
| CL_ETA[13] | -0.14554 | 1.001195 | 4942.662 | 5560.739 | -59.6254 | -0.31525 | 0.018937 | 2.166063 | -7.68544 |
| CL_ETA[14] | -0.07884 | 0.999957 | 5127.622 | 5655.775 | -106.52 | -0.24251 | 0.087902 | 3.076125 | -0.89685 |
| CL_ETA[15] | -0.07257 | 1.000154 | 5414.365 | 4846.138 | -121.236 | -0.24618 | 0.09494 | 3.392164 | -0.7644 |
| CL_ETA[16] | -0.5255 | 1.000796 | 4517.684 | 5693.698 | -15.5545 | -0.69307 | -0.3709 | 1.318882 | 1.41683 |
| CL_ETA[17] | 0.024962 | 1.001825 | 5467.682 | 5102.855 | 343.4175 | -0.14504 | 0.189222 | -5.81036 | 0.131919 |
| CL_ETA[18] | 0.004918 | 1.001206 | 5217.343 | 5303.455 | 1683.607 | -0.16014 | 0.16537 | -32.5629 | 0.029739 |
| CL_ETA[19] | -0.48721 | 1.000317 | 4389.617 | 5333.749 | -16.5481 | -0.6462 | -0.33246 | 1.32633 | 1.465451 |
| CL_ETA[20] | 0.046144 | 1.000581 | 5222.507 | 5454.98 | 190.8783 | -0.13489 | 0.213329 | -2.9233 | 0.216304 |
| CL_ETA[21] | 0.005708 | 1.000182 | 5371.488 | 5628.68 | 1521.791 | -0.16358 | 0.173197 | -28.6566 | 0.032959 |
| CL_ETA[22] | 0.223025 | 1.00023 | 5391.308 | 5250.871 | 40.38438 | 0.044478 | 0.395874 | 0.19943 | 0.563375 |
| CL_ETA[23] | -0.141 | 1.001029 | 5668.244 | 5322.332 | -64.2209 | -0.32406 | 0.029865 | 2.298256 | -4.72141 |
| CL_ETA[24] | 0.163093 | 1.00067 | 5608.953 | 5767.566 | 51.34144 | -0.00276 | 0.324264 | -0.01695 | 0.502964 |
| CL_ETA[25] | -0.05473 | 1.000334 | 5513.256 | 5426.079 | -154.404 | -0.22825 | 0.106938 | 4.170181 | -0.51181 |
| CL_ETA[26] | -0.15389 | 1.000497 | 5219.924 | 5655.863 | -53.0465 | -0.31673 | 0.000983 | 2.058164 | -156.493 |
| CL_ETA[27] | 0.359176 | 0.999756 | 5119.791 | 5614.486 | 25.14484 | 0.177765 | 0.534656 | 0.494924 | 0.67179 |
| CL_ETA[28] | -0.18091 | 1.001787 | 5229.838 | 5480.397 | -45.614 | -0.35345 | -0.02899 | 1.953684 | 6.2416 |
| CL_ETA[29] | 0.278419 | 0.999775 | 5481.641 | 4720.01 | 33.7091 | 0.09643 | 0.463976 | 0.346349 | 0.600071 |
| CL_ETA[30] | 0.020742 | 1.000309 | 4482.979 | 5220.511 | 401.5053 | -0.14682 | 0.183773 | -7.07825 | 0.11287 |
| CL_ETA[31] | 0.115902 | 1.000075 | 4994.62 | 5342.448 | 74.91727 | -0.06123 | 0.278299 | -0.5283 | 0.416464 |
| CL_ETA[32] | -0.17021 | 1.000723 | 4592.061 | 5336.371 | -46.7614 | -0.32996 | -0.01906 | 1.938534 | 8.930044 |
| CL_ETA[33] | -0.18804 | 1.001007 | 4395.364 | 5377.204 | -41.6481 | -0.34196 | -0.03846 | 1.818573 | 4.88958 |
| CL_ETA[34] | 0.09595 | 1.000068 | 4922.09 | 5021.974 | 89.64437 | -0.07669 | 0.260683 | -0.79926 | 0.368073 |
| CL_ETA[35] | -0.17773 | 1.000723 | 5944.166 | 5353.536 | -48.3991 | -0.35831 | -0.022 | 2.016087 | 8.079376 |
| CL_ETA[36] | 0.317072 | 1.000259 | 5800.612 | 5444.293 | 28.53473 | 0.14692 | 0.503266 | 0.463364 | 0.630029 |
| CL_ETA[37] | 0.521876 | 1.000093 | 5570.565 | 5972.963 | 19.17041 | 0.329757 | 0.720306 | 0.631869 | 0.724519 |
| CL_ETA[38] | -0.0744 | 1.000404 | 4326.335 | 4842.725 | -107.127 | -0.2309 | 0.078947 | 3.103389 | -0.94242 |
| CL_ETA[39] | -0.03707 | 1.000464 | 4950.912 | 5100.087 | -226.649 | -0.19774 | 0.130294 | 5.334507 | -0.28449 |
| CL_ETA[40] | -0.12138 | 1.000482 | 4701.508 | 4921.73 | -66.2151 | -0.27484 | 0.041865 | 2.264275 | -2.89937 |
| CL_ETA[41] | 0.045018 | 1.000068 | 5569.451 | 5579.173 | 187.6849 | -0.11528 | 0.217773 | -2.56071 | 0.20672 |
| CL_ETA[42] | 0.157703 | 1.000417 | 4829.277 | 4891.091 | 52.44127 | -0.00845 | 0.318849 | -0.05359 | 0.494601 |
| CL_ETA[43] | 0.162502 | 1.000098 | 5753.856 | 5152 | 53.97379 | -0.00903 | 0.333347 | -0.05558 | 0.487486 |
| CL_ETA[44] | -0.23714 | 1.000271 | 4883.593 | 5429.257 | -36.2447 | -0.40299 | -0.06785 | 1.69935 | 3.495186 |
| CL_ETA[45] | 0.264904 | 1.000189 | 6105.572 | 5499.331 | 34.53698 | 0.08677 | 0.447924 | 0.327552 | 0.591405 |
| CL_ETA[46] | 0.039707 | 1.00033 | 5550.016 | 5320.522 | 221.6612 | -0.13348 | 0.213928 | -3.36156 | 0.185609 |
| CL_ETA[47] | -0.13228 | 1.000272 | 5220.4 | 5014.592 | -68.8216 | -0.31842 | 0.035543 | 2.407084 | -3.7218 |
| CL_ETA[48] | 0.410436 | 1.000169 | 5752.792 | 5654.978 | 22.19938 | 0.222259 | 0.581311 | 0.541518 | 0.706053 |
| CL_ETA[49] | 0.316741 | 1.001263 | 5806.331 | 5767.303 | 28.51458 | 0.139295 | 0.492705 | 0.439778 | 0.64286 |
| CL_ETA[50] | -0.13533 | 1.001279 | 5186.738 | 5549.351 | -63.6607 | -0.31249 | 0.025653 | 2.309011 | -5.27556 |
| V_ETA[1] | -0.0228 | 1.00018 | 5947.729 | 5849.79 | -399.281 | -0.20657 | 0.152555 | 9.060472 | -0.14945 |
| V_ETA[2] | 0.1162 | 1.002328 | 2029.353 | 5622.845 | 93.95171 | -0.08317 | 0.33559 | -0.71575 | 0.346255 |
| V_ETA[3] | 0.093871 | 1.001201 | 2808.185 | 5877.977 | 109.2307 | -0.08876 | 0.304053 | -0.94555 | 0.308732 |
| V_ETA[4] | 0.067458 | 1.001238 | 5478.698 | 5603.081 | 146.32 | -0.11148 | 0.271062 | -1.65263 | 0.248865 |
| V_ETA[5] | 0.013418 | 1.001096 | 8850.579 | 6911.987 | 676.736 | -0.16753 | 0.193551 | -12.4853 | 0.069327 |
| V_ETA[6] | -0.08443 | 1.001465 | 2707.231 | 6065.804 | -110.178 | -0.27328 | 0.08281 | 3.23654 | -1.01963 |
| V_ETA[7] | 0.060369 | 1.000639 | 4688.299 | 6108.138 | 160.0133 | -0.12745 | 0.253588 | -2.11119 | 0.238061 |
| V_ETA[8] | 0.194345 | 1.004216 | 1154.015 | 3578.34 | 71.6996 | -0.06772 | 0.451334 | -0.34844 | 0.4306 |
| V_ETA[9] | -0.15572 | 1.001063 | 1557.414 | 3301.983 | -64.8418 | -0.35394 | 0.031206 | 2.272919 | -4.99017 |
| V_ETA[10] | -0.12089 | 1.002823 | 1570.565 | 4737.167 | -82.0561 | -0.30301 | 0.075197 | 2.506496 | -1.60767 |
| V_ETA[11] | 0.045983 | 1.0008 | 7080.696 | 5581.799 | 208.0407 | -0.12734 | 0.24582 | -2.76926 | 0.18706 |
| V_ETA[12] | 0.006131 | 1.001011 | 9883.077 | 6347.824 | 1454.491 | -0.17463 | 0.18 | -28.4852 | 0.03406 |
| V_ETA[13] | -0.01232 | 1.000471 | 5005.079 | 6072.334 | -744.832 | -0.19127 | 0.171139 | 15.52854 | -0.07197 |
| V_ETA[14] | 0.009458 | 1.000764 | 9541.931 | 5748.345 | 951.8588 | -0.17278 | 0.186572 | -18.2671 | 0.050696 |
| V_ETA[15] | 0.031092 | 1.000043 | 6223.6 | 5518.681 | 303.2406 | -0.15894 | 0.214789 | -5.11172 | 0.144758 |
| V_ETA[16] | -0.25878 | 1.00264 | 1003.185 | 798.0513 | -44.8113 | -0.44562 | 0.005991 | 1.72199 | -43.1923 |
| V_ETA[17] | 0.035401 | 0.999944 | 7648.357 | 6098.79 | 268.5141 | -0.15239 | 0.223345 | -4.30466 | 0.158505 |
| V_ETA[18] | -0.08223 | 1.001795 | 2434.259 | 5639.967 | -121.664 | -0.28357 | 0.10183 | 3.448499 | -0.80754 |
| V_ETA[19] | -0.24002 | 1.003191 | 886.9358 | 984.5815 | -48.6799 | -0.43225 | 0.008412 | 1.800929 | -28.5322 |
| V_ETA[20] | 0.023914 | 1.000255 | 8609.802 | 6051.419 | 398.0457 | -0.1727 | 0.208856 | -7.22144 | 0.114502 |
| V_ETA[21] | 0.063617 | 1.000769 | 4932.224 | 6028.531 | 154.6894 | -0.13266 | 0.253082 | -2.08529 | 0.251368 |
| V_ETA[22] | 0.088841 | 1.001406 | 2823.931 | 5569.032 | 121.5058 | -0.1135 | 0.305685 | -1.27758 | 0.290629 |
| V_ETA[23] | 0.095556 | 1.00173 | 2652.444 | 5380.441 | 104.4729 | -0.09244 | 0.294897 | -0.96738 | 0.324033 |
| V_ETA[24] | -0.09694 | 1.001033 | 1798.119 | 4644.497 | -104.483 | -0.30097 | 0.08667 | 3.10473 | -1.1185 |
| V_ETA[25] | -0.0258 | 1.000106 | 5680.37 | 5241.415 | -353.112 | -0.20543 | 0.155978 | 7.962653 | -0.1654 |
| V_ETA[26] | -0.0612 | 1.000853 | 3074.592 | 5983.789 | -151.227 | -0.25419 | 0.112227 | 4.15317 | -0.54536 |
| V_ETA[27] | 0.030032 | 1.002031 | 6835.723 | 6091.399 | 336.519 | -0.16826 | 0.235147 | -5.60277 | 0.127717 |
| V_ETA[28] | -0.06954 | 1.000658 | 3824.179 | 5792.568 | -131.821 | -0.24858 | 0.11158 | 3.574715 | -0.62323 |
| V_ETA[29] | 0.18535 | 1.003112 | 1221.356 | 3619.596 | 69.75122 | -0.05083 | 0.430656 | -0.27423 | 0.430389 |
| V_ETA[30] | -0.06017 | 1.00141 | 3668.068 | 5986.897 | -160.966 | -0.26482 | 0.111209 | 4.401309 | -0.54104 |
| V_ETA[31] | 0.048001 | 1.000124 | 5686.856 | 6110.344 | 201.7989 | -0.13545 | 0.247819 | -2.82176 | 0.193694 |
| V_ETA[32] | -0.19044 | 1.003804 | 998.2974 | 1548.75 | -58.4079 | -0.40082 | 0.015427 | 2.104694 | -12.3449 |
| V_ETA[33] | -0.17912 | 1.003513 | 978.2445 | 3136.921 | -65.4485 | -0.39744 | 0.036211 | 2.218861 | -4.9466 |
| V_ETA[34] | 0.023329 | 1.000229 | 6890.891 | 6106.038 | 404.7816 | -0.15973 | 0.213508 | -6.847 | 0.109263 |
| V_ETA[35] | -0.04387 | 1.000925 | 6907.096 | 6201.508 | -205.123 | -0.2319 | 0.119502 | 5.286415 | -0.36708 |
| V_ETA[36] | 0.092655 | 1.00133 | 3236.012 | 5662.153 | 117.5034 | -0.11352 | 0.305291 | -1.22515 | 0.303496 |
| V_ETA[37] | 0.224684 | 1.005407 | 1047.893 | 3291.614 | 65.93779 | -0.04507 | 0.497781 | -0.20059 | 0.451371 |
| V_ETA[38] | -0.13987 | 1.002103 | 1490.775 | 4325.443 | -74.8635 | -0.34046 | 0.053436 | 2.434015 | -2.61761 |
| V_ETA[39] | -0.00804 | 0.999819 | 9042.955 | 6574.728 | -1126.92 | -0.19816 | 0.159068 | 24.6342 | -0.05057 |
| V_ETA[40] | -0.14587 | 1.001997 | 1620.218 | 3996.133 | -69.544 | -0.33655 | 0.042847 | 2.307242 | -3.40444 |
| V_ETA[41] | -0.01549 | 1.00137 | 7149.433 | 6067.262 | -599.779 | -0.20627 | 0.164527 | 13.31639 | -0.09415 |
| V_ETA[42] | -0.07815 | 1.001852 | 2338.133 | 5794.004 | -130.239 | -0.28853 | 0.10732 | 3.692102 | -0.72818 |
| V_ETA[43] | 0.040126 | 1.0004 | 8835.769 | 5295.366 | 246.6627 | -0.14885 | 0.241212 | -3.70955 | 0.166351 |
| V_ETA[44] | -0.02176 | 1.000806 | 6147.525 | 5867.444 | -412.429 | -0.2036 | 0.15368 | 9.355978 | -0.1416 |
| V_ETA[45] | 0.141308 | 1.001865 | 1801.427 | 5275.363 | 81.56357 | -0.06788 | 0.369786 | -0.48034 | 0.382135 |
| V_ETA[46] | 0.084667 | 1.001256 | 3379.717 | 6344.717 | 119.1592 | -0.09286 | 0.300217 | -1.09679 | 0.282019 |
| V_ETA[47] | 0.088356 | 1.000961 | 2476.322 | 5110.568 | 111.8566 | -0.09632 | 0.283396 | -1.09014 | 0.311775 |
| V_ETA[48] | 0.022296 | 1.001945 | 9752.527 | 6184.741 | 451.932 | -0.18212 | 0.214003 | -8.1685 | 0.104185 |
| V_ETA[49] | 0.122863 | 1.001681 | 2013.709 | 5989.668 | 92.14673 | -0.08085 | 0.349025 | -0.65801 | 0.352017 |
| V_ETA[50] | 0.023513 | 1.001234 | 8351.003 | 6173.868 | 389.0922 | -0.15904 | 0.206371 | -6.76383 | 0.113937 |

Table S3: individual parameters and random effects estimate for PK model

| Parameters | Median | Rhat | ESS_Bulk | ESS_Tail | RSE | 95CDILB | 95CDIUB | 95CDIRALB | 95CDIRAUB |
| --- | --- | --- | --- | --- | --- | --- | --- | --- | --- |
| A1_IND[1] | 19.27096 | 1.000359 | 7319.272 | 5451.643 | 12.19984 | 15.1167 | 24.21084 | 0.784429 | 0.795964 |
| A1_IND[2] | 16.89596 | 1.001283 | 2256.248 | 3565.781 | 14.4395 | 12.65337 | 21.9736 | 0.748899 | 0.768921 |
| A1_IND[3] | 16.94945 | 1.000674 | 2228.13 | 4260.258 | 14.40717 | 12.8122 | 22.16005 | 0.755907 | 0.764865 |
| A1_IND[4] | 17.31619 | 1.001267 | 2749.005 | 5188.657 | 14.08347 | 13.00444 | 22.35016 | 0.750999 | 0.774768 |
| A1_IND[5] | 18.57358 | 1.0004 | 5108.138 | 6234.408 | 12.3083 | 14.43425 | 23.34506 | 0.777138 | 0.795611 |
| A1_IND[6] | 20.07184 | 0.999899 | 7322.001 | 5868.628 | 12.2023 | 15.82425 | 25.23662 | 0.788381 | 0.795346 |
| A1_IND[7] | 17.59217 | 1.001042 | 3304.827 | 5253.816 | 13.46595 | 13.19951 | 22.38878 | 0.750306 | 0.785758 |
| A1_IND[8] | 15.15956 | 1.001373 | 1214.342 | 2645.269 | 19.06806 | 10.24418 | 21.10626 | 0.675757 | 0.71825 |
| A1_IND[9] | 21.41014 | 1.000736 | 6326.824 | 6170.356 | 12.26273 | 16.89577 | 26.89279 | 0.789148 | 0.796129 |
| A1_IND[10] | 20.70912 | 1.000466 | 6747.967 | 5643.594 | 12.19011 | 16.32617 | 25.94136 | 0.788357 | 0.798305 |
| A1_IND[11] | 17.69527 | 1.000035 | 3192.948 | 5374.061 | 13.52168 | 13.40092 | 22.58422 | 0.757317 | 0.783523 |
| A1_IND[12] | 18.68673 | 1.000305 | 5526.86 | 5847.008 | 12.5192 | 14.35798 | 23.37686 | 0.768352 | 0.799369 |
| A1_IND[13] | 19.01838 | 1.000497 | 6897.726 | 5973.652 | 12.25477 | 14.90377 | 23.97218 | 0.783651 | 0.793352 |
| A1_IND[14] | 18.52643 | 1.000317 | 5517.263 | 5628.418 | 12.66646 | 14.4347 | 23.48736 | 0.779141 | 0.788783 |
| A1_IND[15] | 18.26959 | 1.000886 | 3476.424 | 5866.331 | 12.77125 | 14.13305 | 23.08289 | 0.773583 | 0.791477 |
| A1_IND[16] | 23.53975 | 1.001363 | 1957.543 | 3160.333 | 12.76528 | 18.3197 | 29.76842 | 0.778245 | 0.790762 |
| A1_IND[17] | 17.9553 | 1.000409 | 3896.138 | 5920.088 | 13.25916 | 13.71875 | 22.94279 | 0.76405 | 0.782612 |
| A1_IND[18] | 19.68017 | 1.001011 | 6510.508 | 5708.066 | 12.83095 | 15.2927 | 25.1405 | 0.777062 | 0.782807 |
| A1_IND[19] | 23.15766 | 1.001503 | 2405.79 | 3138.191 | 12.95474 | 17.47392 | 29.01828 | 0.754563 | 0.798037 |
| A1_IND[20] | 18.04586 | 1.00053 | 3866.929 | 4841.485 | 13.20261 | 13.74571 | 22.87491 | 0.76171 | 0.788893 |
| A1_IND[21] | 17.60165 | 1.000806 | 3016.765 | 5228.288 | 13.35988 | 13.4561 | 22.56316 | 0.764479 | 0.780106 |
| A1_IND[22] | 16.88938 | 1.000663 | 2197.373 | 4880.643 | 14.7818 | 12.55065 | 22.15501 | 0.743109 | 0.762328 |
| A1_IND[23] | 17.52207 | 1.000742 | 2897.398 | 4551.028 | 13.33391 | 13.26165 | 22.31998 | 0.756854 | 0.78504 |
| A1_IND[24] | 19.52307 | 1.000576 | 7119.671 | 5932.216 | 13.46609 | 14.73309 | 24.92629 | 0.75465 | 0.783232 |
| A1_IND[25] | 19.04647 | 1.000822 | 6516.985 | 6075.817 | 12.27912 | 14.63973 | 23.71795 | 0.768632 | 0.803041 |
| A1_IND[26] | 19.69862 | 1.001141 | 6988.078 | 6005.244 | 12.20043 | 15.50646 | 24.7249 | 0.787185 | 0.796712 |
| A1_IND[27] | 17.31429 | 1.000391 | 2353.434 | 5603.028 | 14.77262 | 12.71217 | 22.4804 | 0.734201 | 0.770195 |
| A1_IND[28] | 19.83429 | 1.000321 | 7204.519 | 6282.224 | 12.13785 | 15.77524 | 25.0514 | 0.795352 | 0.791744 |
| A1_IND[29] | 15.63994 | 1.000905 | 1312.339 | 3174.333 | 16.98786 | 11.26755 | 21.33629 | 0.720434 | 0.733021 |
| A1_IND[30] | 19.3418 | 1.000117 | 7199.85 | 6010.297 | 12.72614 | 15.04745 | 24.58927 | 0.777976 | 0.786595 |
| A1_IND[31] | 17.5505 | 1.002105 | 2983.095 | 4544.111 | 13.88649 | 13.34536 | 22.6618 | 0.760398 | 0.774453 |
| A1_IND[32] | 21.64315 | 1.00135 | 4204.43 | 5580.843 | 12.85994 | 16.80333 | 27.44445 | 0.776381 | 0.788617 |
| A1_IND[33] | 21.62452 | 1.001 | 4133.773 | 5123.59 | 13.23537 | 16.7969 | 27.7125 | 0.776753 | 0.780317 |
| A1_IND[34] | 17.9594 | 1.000197 | 3601.316 | 5716.55 | 13.45848 | 13.69041 | 22.84354 | 0.762298 | 0.786191 |
| A1_IND[35] | 19.50898 | 1.000146 | 7046.05 | 6100.922 | 12.05576 | 15.5335 | 24.53253 | 0.796223 | 0.795229 |
| A1_IND[36] | 16.64241 | 1.00072 | 1809.599 | 4678.842 | 15.53572 | 12.09405 | 21.836 | 0.726701 | 0.762155 |
| A1_IND[37] | 14.83486 | 1.001593 | 1064.392 | 2518.926 | 19.88854 | 9.756442 | 20.6962 | 0.65767 | 0.716792 |
| A1_IND[38] | 20.64387 | 1.000331 | 6752.644 | 5949.024 | 12.79607 | 16.2156 | 26.09656 | 0.785492 | 0.791057 |
| A1_IND[39] | 18.70208 | 1.000155 | 6663.464 | 5479.663 | 12.68976 | 14.6592 | 23.77058 | 0.783827 | 0.786774 |
| A1_IND[40] | 20.79098 | 1.001191 | 6205.438 | 5657.368 | 12.29538 | 16.35752 | 26.20546 | 0.786761 | 0.793383 |
| A1_IND[41] | 18.64228 | 1.000083 | 5975.519 | 5879.109 | 12.88181 | 14.38136 | 23.69044 | 0.771438 | 0.786911 |
| A1_IND[42] | 19.35116 | 1.000135 | 6852.887 | 5934.488 | 13.32254 | 14.94465 | 25.012 | 0.772287 | 0.773675 |
| A1_IND[43] | 17.5826 | 1.00043 | 3316.274 | 5174.97 | 13.74956 | 13.30415 | 22.55955 | 0.756666 | 0.779386 |
| A1_IND[44] | 19.287 | 1.000446 | 6529.076 | 6143.77 | 11.97682 | 15.10547 | 24.1267 | 0.783194 | 0.799405 |
| A1_IND[45] | 16.15292 | 1.001668 | 1550.096 | 3778.158 | 15.90642 | 11.7923 | 21.60611 | 0.730042 | 0.747609 |
| A1_IND[46] | 17.28278 | 1.000455 | 2455.833 | 4650.159 | 13.91045 | 12.89662 | 22.08052 | 0.746212 | 0.782716 |
| A1_IND[47] | 17.55953 | 1.000872 | 2733.107 | 4627.274 | 13.45645 | 13.5107 | 22.65088 | 0.769423 | 0.775225 |
| A1_IND[48] | 17.37571 | 1.000042 | 2829.754 | 5508.274 | 14.85992 | 12.73819 | 22.55256 | 0.733103 | 0.770454 |
| A1_IND[49] | 16.23084 | 1.000493 | 1608.98 | 4108.882 | 15.90637 | 11.68269 | 21.56368 | 0.719784 | 0.752693 |
| A1_IND[50] | 18.44249 | 1.000523 | 4898.871 | 5879.966 | 12.60538 | 14.17148 | 23.11683 | 0.768414 | 0.797795 |
| B1_IND[1] | 0.111891 | 1.000212 | 8225.555 | 6073.389 | 7.759624 | 0.095372 | 0.129185 | 0.852364 | 0.866132 |
| B1_IND[2] | 0.121363 | 1.0005 | 4546.796 | 5933.611 | 8.132032 | 0.103135 | 0.141769 | 0.849807 | 0.856061 |
| B1_IND[3] | 0.128041 | 1.000953 | 4236.061 | 5569.325 | 8.209682 | 0.107572 | 0.148751 | 0.840138 | 0.860776 |
| B1_IND[4] | 0.129305 | 1.000885 | 4311.746 | 5507.538 | 8.118087 | 0.108661 | 0.149744 | 0.84035 | 0.863509 |
| B1_IND[5] | 0.114299 | 1.000365 | 8565.62 | 6470.986 | 7.751307 | 0.096934 | 0.131592 | 0.848072 | 0.868586 |
| B1_IND[6] | 0.116503 | 0.999899 | 8516.819 | 6395.277 | 7.593502 | 0.099747 | 0.134472 | 0.856179 | 0.866372 |
| B1_IND[7] | 0.122391 | 1.000126 | 5818.642 | 6192.053 | 7.917738 | 0.103523 | 0.141542 | 0.845836 | 0.8647 |
| B1_IND[8] | 0.14788 | 1.000935 | 1959.967 | 4261.589 | 9.402048 | 0.120581 | 0.173894 | 0.815398 | 0.850402 |
| B1_IND[9] | 0.111253 | 1.000502 | 7224.021 | 6378.063 | 8.009988 | 0.094117 | 0.129163 | 0.845976 | 0.861334 |
| B1_IND[10] | 0.11487 | 1.000492 | 8217.126 | 6109.458 | 7.69878 | 0.09786 | 0.13257 | 0.851919 | 0.866489 |
| B1_IND[11] | 0.124844 | 1.000154 | 5915.074 | 5871.474 | 8.017223 | 0.104767 | 0.143857 | 0.839181 | 0.867837 |
| B1_IND[12] | 0.115335 | 0.999906 | 8745.994 | 5599.516 | 7.923579 | 0.097844 | 0.133628 | 0.848348 | 0.863103 |
| B1_IND[13] | 0.115875 | 1.000377 | 8033.521 | 6616.598 | 7.773978 | 0.098937 | 0.133999 | 0.853832 | 0.864745 |
| B1_IND[14] | 0.117966 | 1.000214 | 7452.658 | 6208.406 | 7.769258 | 0.100212 | 0.135572 | 0.849499 | 0.870139 |
| B1_IND[15] | 0.117094 | 1.000536 | 6657.405 | 6188.999 | 7.838628 | 0.098823 | 0.134855 | 0.843965 | 0.868292 |
| B1_IND[16] | 0.103126 | 1.00108 | 2621.331 | 4711.269 | 8.671474 | 0.086336 | 0.121328 | 0.83719 | 0.849975 |
| B1_IND[17] | 0.123697 | 1.000341 | 6911.049 | 6110.53 | 7.875694 | 0.104568 | 0.142454 | 0.845359 | 0.868329 |
| B1_IND[18] | 0.129819 | 1.000567 | 8083.449 | 5805.946 | 7.809899 | 0.110599 | 0.15046 | 0.851947 | 0.862809 |
| B1_IND[19] | 0.105101 | 1.001051 | 3066.134 | 5280.355 | 8.457507 | 0.088256 | 0.122817 | 0.83973 | 0.855752 |
| B1_IND[20] | 0.125492 | 1.000398 | 6784.851 | 6061.866 | 7.823758 | 0.105964 | 0.144179 | 0.844388 | 0.870395 |
| B1_IND[21] | 0.120816 | 1.000315 | 6051.045 | 6526.794 | 8.052657 | 0.101758 | 0.139856 | 0.842262 | 0.863859 |
| B1_IND[22] | 0.133755 | 1.001027 | 3864.425 | 5658.273 | 8.28441 | 0.112648 | 0.155968 | 0.8422 | 0.857581 |
| B1_IND[23] | 0.109916 | 1.000435 | 6713.072 | 5833.814 | 8.242381 | 0.093052 | 0.128121 | 0.846567 | 0.85791 |
| B1_IND[24] | 0.141734 | 1.000258 | 7051.863 | 6074.188 | 7.97404 | 0.119753 | 0.163661 | 0.844911 | 0.866023 |
| B1_IND[25] | 0.122374 | 1.00026 | 8448.347 | 6228.47 | 7.591071 | 0.10412 | 0.140195 | 0.85083 | 0.872882 |
| B1_IND[26] | 0.117562 | 1.000219 | 8185.223 | 6347.567 | 7.636087 | 0.099982 | 0.135283 | 0.850463 | 0.869005 |
| B1_IND[27] | 0.146781 | 1.0004 | 2892.08 | 5836.757 | 8.581795 | 0.121926 | 0.170291 | 0.830664 | 0.861941 |
| B1_IND[28] | 0.115674 | 1.000104 | 8604.396 | 5677.683 | 7.762653 | 0.098825 | 0.134092 | 0.85434 | 0.862649 |
| B1_IND[29] | 0.133014 | 0.999921 | 2476.67 | 5946.571 | 8.625707 | 0.110268 | 0.154863 | 0.828991 | 0.858917 |
| B1_IND[30] | 0.128738 | 1.00021 | 8504.762 | 6429.447 | 7.769428 | 0.109986 | 0.149064 | 0.854342 | 0.863645 |
| B1_IND[31] | 0.128669 | 1.00155 | 4429.377 | 5735.68 | 8.093645 | 0.109031 | 0.15007 | 0.847373 | 0.857399 |
| B1_IND[32] | 0.123274 | 1.000289 | 7633.082 | 6139.282 | 7.707796 | 0.103986 | 0.140805 | 0.843534 | 0.8755 |
| B1_IND[33] | 0.122127 | 1.000074 | 8571.238 | 5897.808 | 7.701128 | 0.104432 | 0.141359 | 0.85511 | 0.863952 |
| B1_IND[34] | 0.128559 | 0.999809 | 5231.894 | 6364.807 | 7.84105 | 0.10867 | 0.147405 | 0.845292 | 0.87215 |
| B1_IND[35] | 0.11447 | 0.999923 | 8732.148 | 6200.901 | 7.810919 | 0.096755 | 0.131823 | 0.845246 | 0.86836 |
| B1_IND[36] | 0.140687 | 1.000186 | 2557.905 | 5450.473 | 8.515947 | 0.117928 | 0.164531 | 0.838227 | 0.85508 |
| B1_IND[37] | 0.14809 | 1.001106 | 1897.158 | 4670.601 | 9.49212 | 0.122319 | 0.176155 | 0.825979 | 0.84068 |
| B1_IND[38] | 0.126971 | 1.000211 | 9965.559 | 6528.759 | 7.716389 | 0.10868 | 0.147227 | 0.855946 | 0.862416 |
| B1_IND[39] | 0.121994 | 1.000088 | 7918.529 | 6080.829 | 7.740828 | 0.104205 | 0.140982 | 0.854181 | 0.865313 |
| B1_IND[40] | 0.123464 | 1.000525 | 8921.521 | 5632.959 | 7.558722 | 0.105138 | 0.141478 | 0.851566 | 0.872673 |
| B1_IND[41] | 0.12845 | 0.999951 | 7485.687 | 5721.968 | 7.881166 | 0.107725 | 0.147909 | 0.838654 | 0.868439 |
| B1_IND[42] | 0.140179 | 0.99985 | 6173.678 | 5218.212 | 8.05812 | 0.118138 | 0.162182 | 0.842762 | 0.864332 |
| B1_IND[43] | 0.133549 | 1.00049 | 4270.973 | 5643.237 | 8.077033 | 0.111598 | 0.154182 | 0.835639 | 0.866177 |
| B1_IND[44] | 0.109857 | 1.001077 | 7833.484 | 6321.209 | 7.896121 | 0.093834 | 0.127676 | 0.854144 | 0.860434 |
| B1_IND[45] | 0.134569 | 1.001077 | 2671.045 | 4843.361 | 8.507846 | 0.11328 | 0.157194 | 0.841797 | 0.856071 |
| B1_IND[46] | 0.121762 | 1.000139 | 5578.361 | 6493.695 | 8.016218 | 0.102646 | 0.140578 | 0.843004 | 0.866154 |
| B1_IND[47] | 0.110734 | 0.999817 | 6554.38 | 5999.93 | 8.237129 | 0.092495 | 0.128025 | 0.835297 | 0.864938 |
| B1_IND[48] | 0.15174 | 1.000664 | 2564.939 | 5163.967 | 8.688214 | 0.127181 | 0.177952 | 0.838152 | 0.852698 |
| B1_IND[49] | 0.138899 | 0.999867 | 2581.905 | 5543.951 | 8.478068 | 0.116215 | 0.161911 | 0.836689 | 0.857875 |
| B1_IND[50] | 0.113482 | 1.000064 | 8440.437 | 6237.824 | 8.091672 | 0.095084 | 0.130933 | 0.837871 | 0.866723 |
| B2_IND[1] | 0.345061 | 1.000587 | 7383.726 | 5324.439 | 7.59452 | 0.2945 | 0.399196 | 0.853472 | 0.864389 |
| B2_IND[2] | 0.331045 | 1.000397 | 2032.513 | 3696.393 | 8.423499 | 0.273187 | 0.382368 | 0.825227 | 0.865776 |
| B2_IND[3] | 0.33728 | 1.000555 | 2706.904 | 4075.457 | 7.95529 | 0.283278 | 0.387623 | 0.83989 | 0.870123 |
| B2_IND[4] | 0.341011 | 1.000202 | 3571.6 | 4499.348 | 7.739909 | 0.288233 | 0.391889 | 0.845231 | 0.870172 |
| B2_IND[5] | 0.335974 | 1.000898 | 3157.733 | 4934.807 | 7.940424 | 0.284252 | 0.387703 | 0.846054 | 0.866576 |
| B2_IND[6] | 0.349973 | 1.00108 | 8441.647 | 6123.567 | 7.819214 | 0.299051 | 0.405071 | 0.854497 | 0.863981 |
| B2_IND[7] | 0.336627 | 1.000651 | 3069.076 | 4284.569 | 7.917827 | 0.285434 | 0.389441 | 0.847924 | 0.864386 |
| B2_IND[8] | 0.332007 | 1.00108 | 1748.27 | 3003.342 | 8.674684 | 0.272614 | 0.385104 | 0.821112 | 0.862122 |
| B2_IND[9] | 0.347916 | 1.000799 | 9235.388 | 6021.992 | 7.501867 | 0.29896 | 0.400491 | 0.859288 | 0.868723 |
| B2_IND[10] | 0.355281 | 1.000815 | 7567.161 | 6144.391 | 7.957642 | 0.30517 | 0.415247 | 0.858954 | 0.855591 |
| B2_IND[11] | 0.33974 | 1.000444 | 3483.332 | 4740.653 | 7.734081 | 0.287544 | 0.390714 | 0.846364 | 0.869538 |
| B2_IND[12] | 0.339023 | 1.000521 | 4516.317 | 4517.165 | 7.76208 | 0.28757 | 0.391067 | 0.848232 | 0.866917 |
| B2_IND[13] | 0.350219 | 1.000036 | 8127.073 | 6749.512 | 7.909637 | 0.30231 | 0.411671 | 0.863202 | 0.850727 |
| B2_IND[14] | 0.34063 | 1.000172 | 5125.295 | 4777.381 | 7.903304 | 0.286396 | 0.39194 | 0.840783 | 0.869087 |
| B2_IND[15] | 0.333397 | 1.000517 | 2604.457 | 3977.575 | 8.137169 | 0.276879 | 0.382675 | 0.830477 | 0.871229 |
| B2_IND[16] | 0.353722 | 1.000777 | 4911.426 | 5191.45 | 8.042654 | 0.302545 | 0.412096 | 0.85532 | 0.858347 |
| B2_IND[17] | 0.341886 | 1.00039 | 5081.963 | 5069.352 | 7.57867 | 0.288248 | 0.391261 | 0.843113 | 0.873805 |
| B2_IND[18] | 0.360309 | 1.00071 | 6571.785 | 6286.295 | 7.96684 | 0.313078 | 0.423141 | 0.868915 | 0.851511 |
| B2_IND[19] | 0.357759 | 1.000997 | 3977.218 | 3147.523 | 8.408467 | 0.306291 | 0.42126 | 0.856138 | 0.849261 |
| B2_IND[20] | 0.340649 | 1.001327 | 4744.335 | 4802.215 | 7.764862 | 0.286139 | 0.391298 | 0.839984 | 0.870561 |
| B2_IND[21] | 0.335653 | 1.000438 | 2395.474 | 4265.098 | 7.915595 | 0.283482 | 0.387653 | 0.84457 | 0.865858 |
| B2_IND[22] | 0.336103 | 1.000643 | 2692.063 | 3684.437 | 7.972482 | 0.284274 | 0.390505 | 0.845794 | 0.860689 |
| B2_IND[23] | 0.329053 | 1.001273 | 1890.834 | 3859.685 | 8.772648 | 0.270536 | 0.38345 | 0.822165 | 0.858138 |
| B2_IND[24] | 0.363688 | 1.000487 | 5240.121 | 5644.717 | 8.539509 | 0.313113 | 0.430337 | 0.860938 | 0.845123 |
| B2_IND[25] | 0.348148 | 1.000423 | 7963.76 | 5922.407 | 7.697873 | 0.296621 | 0.40149 | 0.851995 | 0.867141 |
| B2_IND[26] | 0.350775 | 1.000741 | 8821.221 | 6320.753 | 7.714661 | 0.300046 | 0.4067 | 0.855381 | 0.862489 |
| B2_IND[27] | 0.346111 | 1.00006 | 6439.581 | 5710.941 | 7.666978 | 0.296151 | 0.399879 | 0.855654 | 0.865538 |
| B2_IND[28] | 0.346957 | 1.000587 | 8516.741 | 6291.2 | 7.533951 | 0.298479 | 0.400753 | 0.860277 | 0.865763 |
| B2_IND[29] | 0.329083 | 1.001044 | 1604.313 | 2835.569 | 8.824376 | 0.266592 | 0.378898 | 0.810107 | 0.868527 |
| B2_IND[30] | 0.355078 | 1.000208 | 8105.275 | 6284.895 | 7.931678 | 0.303583 | 0.412421 | 0.854977 | 0.86096 |
| B2_IND[31] | 0.339622 | 1.000912 | 3731.891 | 5230.96 | 7.743518 | 0.28991 | 0.393887 | 0.853627 | 0.862233 |
| B2_IND[32] | 0.3646 | 1.001129 | 3740.776 | 5801.719 | 8.675716 | 0.313718 | 0.435121 | 0.860444 | 0.837928 |
| B2_IND[33] | 0.369893 | 1.001274 | 2816.13 | 5578.848 | 9.549132 | 0.315136 | 0.44703 | 0.851966 | 0.827447 |
| B2_IND[34] | 0.341402 | 1.000817 | 4637.911 | 5018.561 | 7.617942 | 0.291198 | 0.393534 | 0.852948 | 0.867529 |
| B2_IND[35] | 0.342918 | 0.999913 | 7472.086 | 6527.834 | 7.483522 | 0.293605 | 0.395317 | 0.856195 | 0.86745 |
| B2_IND[36] | 0.341308 | 1.000976 | 3311.652 | 4225.583 | 7.683855 | 0.288483 | 0.391478 | 0.845227 | 0.871845 |
| B2_IND[37] | 0.325436 | 1.001326 | 1304.694 | 3203.823 | 9.131272 | 0.264326 | 0.377994 | 0.812222 | 0.860956 |
| B2_IND[38] | 0.361475 | 1.000657 | 4884.298 | 5284.055 | 8.342866 | 0.312176 | 0.426753 | 0.863618 | 0.847036 |
| B2_IND[39] | 0.346249 | 0.999932 | 7431.346 | 6213.202 | 7.59892 | 0.296135 | 0.398341 | 0.855265 | 0.869227 |
| B2_IND[40] | 0.355753 | 1.000884 | 7025.354 | 5900.667 | 7.850383 | 0.307865 | 0.41621 | 0.865389 | 0.854745 |
| B2_IND[41] | 0.35087 | 1.000176 | 8444.92 | 6452.195 | 7.541746 | 0.302638 | 0.40587 | 0.862537 | 0.864488 |
| B2_IND[42] | 0.362908 | 1.001003 | 5159.469 | 6012.078 | 8.41591 | 0.312258 | 0.429748 | 0.860434 | 0.844467 |
| B2_IND[43] | 0.349343 | 0.999934 | 7474.94 | 6602.716 | 7.642273 | 0.300219 | 0.405057 | 0.85938 | 0.862454 |
| B2_IND[44] | 0.343671 | 1.000428 | 6974.099 | 5973.207 | 7.706845 | 0.294413 | 0.399363 | 0.85667 | 0.860547 |
| B2_IND[45] | 0.335888 | 1.000911 | 2135.849 | 3575.781 | 8.17436 | 0.279524 | 0.387839 | 0.832193 | 0.866051 |
| B2_IND[46] | 0.33353 | 1.000262 | 2309.445 | 3580.87 | 8.322576 | 0.27694 | 0.384647 | 0.830329 | 0.867107 |
| B2_IND[47] | 0.328539 | 1.001092 | 1796.888 | 3362.053 | 8.741681 | 0.2679 | 0.379412 | 0.815427 | 0.865918 |
| B2_IND[48] | 0.353144 | 1.001086 | 7497.459 | 6637.82 | 7.593592 | 0.306853 | 0.411686 | 0.868917 | 0.857799 |
| B2_IND[49] | 0.33759 | 1.000428 | 2665.466 | 3336.71 | 7.963483 | 0.283929 | 0.389604 | 0.841049 | 0.866494 |
| B2_IND[50] | 0.335509 | 1.000455 | 3427.089 | 4579.272 | 7.899491 | 0.284496 | 0.385777 | 0.847955 | 0.869696 |
| A1_ETA[1] | 0.042427 | 1.000062 | 5755.324 | 6555.096 | 226.7144 | -0.13728 | 0.236652 | -3.23567 | 0.179278 |
| A1_ETA[2] | -0.08369 | 1.000923 | 4726.575 | 6174.791 | -127.828 | -0.30414 | 0.111005 | 3.634247 | -0.75391 |
| A1_ETA[3] | -0.07869 | 1.000287 | 5157.752 | 6515.715 | -132.615 | -0.29626 | 0.109302 | 3.764719 | -0.71997 |
| A1_ETA[4] | -0.05909 | 1.000232 | 6278.236 | 6235.197 | -175.918 | -0.2685 | 0.137286 | 4.543703 | -0.43043 |
| A1_ETA[5] | 0.005262 | 1.000128 | 9473.267 | 6584.662 | 1816.162 | -0.18284 | 0.195901 | -34.7471 | 0.02686 |
| A1_ETA[6] | 0.083979 | 1.000548 | 3201.67 | 5014.224 | 119.5768 | -0.09321 | 0.298017 | -1.10996 | 0.281791 |
| A1_ETA[7] | -0.04269 | 1.000402 | 7069.142 | 6098.022 | -232.374 | -0.243 | 0.152155 | 5.69288 | -0.28054 |
| A1_ETA[8] | -0.19167 | 1.000921 | 1396.662 | 4314.669 | -76.3052 | -0.49319 | 0.052091 | 2.573099 | -3.67955 |
| A1_ETA[9] | 0.150187 | 1.000902 | 1777.347 | 5275.617 | 71.44352 | -0.04363 | 0.366629 | -0.29052 | 0.409644 |
| A1_ETA[10] | 0.118124 | 1.00069 | 1932.05 | 5869.11 | 88.83324 | -0.07333 | 0.331454 | -0.62076 | 0.356381 |
| A1_ETA[11] | -0.03715 | 1.00082 | 8205.833 | 6506.04 | -271.741 | -0.2403 | 0.156921 | 6.46887 | -0.23673 |
| A1_ETA[12] | 0.00892 | 1.000193 | 7862.272 | 5759.247 | 1079.098 | -0.18007 | 0.199989 | -20.1871 | 0.044604 |
| A1_ETA[13] | 0.029778 | 1.000471 | 6737.105 | 5716.989 | 323.2161 | -0.15414 | 0.22253 | -5.17642 | 0.133816 |
| A1_ETA[14] | 0.004818 | 1.001632 | 9320.177 | 6121.511 | 2019.112 | -0.19232 | 0.192092 | -39.9198 | 0.025079 |
| A1_ETA[15] | -0.00748 | 1.000662 | 7723.685 | 6012.352 | -1303.33 | -0.20289 | 0.186009 | 27.11124 | -0.04023 |
| A1_ETA[16] | 0.250424 | 1.001809 | 927.2814 | 845.1257 | 49.10401 | -0.00466 | 0.457957 | -0.01863 | 0.54683 |
| A1_ETA[17] | -0.02416 | 1.00133 | 8973.938 | 6041.274 | -402.757 | -0.21158 | 0.172323 | 8.756176 | -0.14022 |
| A1_ETA[18] | 0.064289 | 1.000718 | 4270.537 | 5661.136 | 161.8371 | -0.12817 | 0.278062 | -1.99372 | 0.231205 |
| A1_ETA[19] | 0.234066 | 1.001864 | 1053.676 | 800.3222 | 52.19519 | -0.00788 | 0.450261 | -0.03365 | 0.519845 |
| A1_ETA[20] | -0.01883 | 1.000723 | 8181.367 | 5470.149 | -517.503 | -0.2089 | 0.174815 | 11.09247 | -0.10773 |
| A1_ETA[21] | -0.04013 | 0.99991 | 7100.641 | 5940.879 | -245.903 | -0.2539 | 0.137898 | 6.326693 | -0.29102 |
| A1_ETA[22] | -0.08232 | 1.000517 | 3945.684 | 6460.032 | -134.646 | -0.30194 | 0.124905 | 3.668008 | -0.65905 |
| A1_ETA[23] | -0.04622 | 0.999864 | 7320.952 | 5893.84 | -218.225 | -0.24881 | 0.151422 | 5.382692 | -0.30527 |
| A1_ETA[24] | 0.05571 | 1.000404 | 3981.282 | 5638.972 | 196.6416 | -0.1434 | 0.286987 | -2.57396 | 0.194121 |
| A1_ETA[25] | 0.029376 | 1.000106 | 7422.328 | 6070.513 | 326.4434 | -0.14885 | 0.234198 | -5.06712 | 0.125432 |
| A1_ETA[26] | 0.063435 | 1.001051 | 3978.823 | 5643.786 | 154.4006 | -0.11625 | 0.274797 | -1.83265 | 0.230843 |
| A1_ETA[27] | -0.05801 | 1.000047 | 4870.783 | 6165.894 | -191.646 | -0.28294 | 0.154116 | 4.87781 | -0.37637 |
| A1_ETA[28] | 0.070921 | 0.99988 | 3323.303 | 5607.878 | 137.8125 | -0.11046 | 0.267819 | -1.55749 | 0.264808 |
| A1_ETA[29] | -0.15751 | 1.000603 | 1675.711 | 4423.405 | -80.1081 | -0.39614 | 0.083345 | 2.514975 | -1.88989 |
| A1_ETA[30] | 0.044386 | 1.000159 | 5251.833 | 5913.094 | 223.6194 | -0.14719 | 0.245759 | -3.31606 | 0.180607 |
| A1_ETA[31] | -0.04331 | 1.000725 | 6833.77 | 5743.14 | -233.51 | -0.25155 | 0.143169 | 5.808699 | -0.30249 |
| A1_ETA[32] | 0.16012 | 1.00135 | 1457.963 | 4003.247 | 71.26034 | -0.03671 | 0.390761 | -0.22927 | 0.409765 |
| A1_ETA[33] | 0.161955 | 1.001278 | 1366.049 | 4230.986 | 71.86599 | -0.03775 | 0.399184 | -0.23308 | 0.405715 |
| A1_ETA[34] | -0.02336 | 1.000761 | 7711.449 | 6018.311 | -427.721 | -0.22009 | 0.170893 | 9.422128 | -0.13669 |
| A1_ETA[35] | 0.052999 | 1.000696 | 4652.605 | 5286.544 | 183.2133 | -0.12627 | 0.260824 | -2.38248 | 0.203197 |
| A1_ETA[36] | -0.09746 | 1.00022 | 2707.706 | 5626.533 | -118.819 | -0.32645 | 0.119898 | 3.349703 | -0.81282 |
| A1_ETA[37] | -0.21075 | 1.001898 | 1193.037 | 5152.624 | -73.5294 | -0.50793 | 0.059859 | 2.410141 | -3.52074 |
| A1_ETA[38] | 0.113573 | 1.00069 | 2162.558 | 5538.613 | 94.58468 | -0.08198 | 0.330158 | -0.72182 | 0.343998 |
| A1_ETA[39] | 0.014312 | 1.000051 | 9457.287 | 5690.96 | 673.0145 | -0.1595 | 0.220135 | -11.1446 | 0.065015 |
| A1_ETA[40] | 0.120881 | 1.001117 | 1910.398 | 5463.332 | 86.21303 | -0.05817 | 0.339099 | -0.48126 | 0.356477 |
| A1_ETA[41] | 0.009289 | 1.000567 | 8907.713 | 5850.034 | 1065.242 | -0.17408 | 0.222052 | -18.74 | 0.041833 |
| A1_ETA[42] | 0.044396 | 1.000915 | 5028.358 | 5721.411 | 238.8725 | -0.15466 | 0.270175 | -3.48371 | 0.164322 |
| A1_ETA[43] | -0.04416 | 1.000297 | 8296.475 | 6268.391 | -231.907 | -0.24658 | 0.157328 | 5.584282 | -0.28066 |
| A1_ETA[44] | 0.040784 | 1.000351 | 5657.035 | 5685.667 | 238.0465 | -0.13693 | 0.248703 | -3.35751 | 0.163988 |
| A1_ETA[45] | -0.12712 | 1.00101 | 2147.869 | 4874.486 | -92.959 | -0.36101 | 0.090308 | 2.839867 | -1.40765 |
| A1_ETA[46] | -0.05954 | 1.000124 | 5580.313 | 5465.925 | -172.492 | -0.27239 | 0.134777 | 4.574682 | -0.44179 |
| A1_ETA[47] | -0.04412 | 1.000152 | 5684.693 | 5504.662 | -232.32 | -0.25412 | 0.14426 | 5.759322 | -0.30585 |
| A1_ETA[48] | -0.05721 | 1.000286 | 6508.389 | 6013.592 | -197.996 | -0.29908 | 0.147716 | 5.227705 | -0.3873 |
| A1_ETA[49] | -0.12019 | 1.000697 | 2437.14 | 6107.213 | -97.2714 | -0.34811 | 0.098454 | 2.896302 | -1.22077 |
| A1_ETA[50] | 0.000103 | 1.001032 | 9001.372 | 6251.277 | 96087.56 | -0.19918 | 0.196973 | -1929.48 | 0.000524 |
| B1_ETA[1] | -0.10231 | 0.999942 | 7188.871 | 6425.163 | -68.8981 | -0.24503 | 0.030768 | 2.39505 | -3.32521 |
| B1_ETA[2] | -0.02101 | 0.999844 | 8589.659 | 6071.485 | -338.078 | -0.17327 | 0.106173 | 8.246331 | -0.1979 |
| B1_ETA[3] | 0.030875 | 0.999949 | 8464.642 | 5753.345 | 232.1322 | -0.11382 | 0.165994 | -3.68639 | 0.186001 |
| B1_ETA[4] | 0.040063 | 1.000376 | 8335.834 | 6133.41 | 180.7881 | -0.10498 | 0.177563 | -2.62046 | 0.225629 |
| B1_ETA[5] | -0.08236 | 1.000952 | 9586.747 | 6187.983 | -85.9383 | -0.22745 | 0.047889 | 2.761591 | -1.71984 |
| B1_ETA[6] | -0.06227 | 1.000198 | 6591.957 | 5933.12 | -113.691 | -0.20855 | 0.07553 | 3.34917 | -0.82442 |
| B1_ETA[7] | -0.01386 | 0.999859 | 9475.286 | 6545.172 | -508.426 | -0.15287 | 0.121055 | 11.02648 | -0.11452 |
| B1_ETA[8] | 0.174294 | 1.000627 | 2735.13 | 4157.06 | 48.3914 | 0.009608 | 0.332644 | 0.055126 | 0.523966 |
| B1_ETA[9] | -0.11001 | 1.000574 | 3285.329 | 3996.743 | -70.2703 | -0.26678 | 0.033628 | 2.425054 | -3.27138 |
| B1_ETA[10] | -0.07821 | 1.000389 | 4174.853 | 6013.884 | -93.3397 | -0.22788 | 0.060772 | 2.913862 | -1.28688 |
| B1_ETA[11] | 0.005895 | 1.000612 | 9322.017 | 6053.812 | 1206.107 | -0.13917 | 0.138881 | -23.6105 | 0.042443 |
| B1_ETA[12] | -0.07438 | 0.999925 | 9766.407 | 6497.932 | -96.4369 | -0.21537 | 0.066272 | 2.895526 | -1.12234 |
| B1_ETA[13] | -0.06725 | 1.000687 | 8376.835 | 6143.882 | -103.91 | -0.20651 | 0.07072 | 3.070796 | -0.95092 |
| B1_ETA[14] | -0.05135 | 1.000826 | 9690.191 | 5520.718 | -136.332 | -0.18774 | 0.086303 | 3.656403 | -0.59495 |
| B1_ETA[15] | -0.05856 | 1.000679 | 8964.521 | 5954.831 | -122.701 | -0.20342 | 0.076497 | 3.473672 | -0.76552 |
| B1_ETA[16] | -0.18203 | 1.001027 | 1621.62 | 3201.799 | -47.0353 | -0.36582 | -0.0333 | 2.009672 | 5.46688 |
| B1_ETA[17] | -0.00256 | 1.000369 | 9983.397 | 6326.986 | -2706.6 | -0.14601 | 0.123548 | 57.01432 | -0.02073 |
| B1_ETA[18] | 0.044047 | 1.000015 | 9752.847 | 5995.658 | 162.5475 | -0.09622 | 0.183128 | -2.18454 | 0.240524 |
| B1_ETA[19] | -0.16527 | 1.000968 | 1849.253 | 3878.429 | -49.473 | -0.32826 | -0.01475 | 1.986176 | 11.20701 |
| B1_ETA[20] | 0.011295 | 1.000753 | 10738.82 | 6257.836 | 625.4491 | -0.13381 | 0.14225 | -11.8462 | 0.079405 |
| B1_ETA[21] | -0.02711 | 0.999991 | 10385.63 | 6421.977 | -262.096 | -0.16964 | 0.110463 | 6.257332 | -0.24543 |
| B1_ETA[22] | 0.073007 | 1.001209 | 7063.804 | 5911.947 | 103.1569 | -0.07154 | 0.220608 | -0.97992 | 0.330936 |
| B1_ETA[23] | -0.1206 | 1.000249 | 8685.718 | 6317.079 | -61.6726 | -0.26385 | 0.028446 | 2.187828 | -4.23954 |
| B1_ETA[24] | 0.130156 | 1.000954 | 9260.361 | 5966.119 | 58.11584 | -0.01877 | 0.280422 | -0.14418 | 0.464146 |
| B1_ETA[25] | -0.01513 | 0.999907 | 9865.993 | 6395.765 | -457.62 | -0.15015 | 0.120618 | 9.925964 | -0.12541 |
| B1_ETA[26] | -0.05437 | 1.000335 | 7470.159 | 6427.068 | -129.021 | -0.2007 | 0.074092 | 3.691096 | -0.73386 |
| B1_ETA[27] | 0.165501 | 1.000669 | 5238.874 | 5528.687 | 48.10278 | 0.006209 | 0.31454 | 0.037517 | 0.526167 |
| B1_ETA[28] | -0.07117 | 0.999975 | 6715.745 | 5688.588 | -100.581 | -0.21521 | 0.064308 | 3.02399 | -1.10666 |
| B1_ETA[29] | 0.06843 | 0.999748 | 4279.645 | 5737.432 | 110.7565 | -0.08869 | 0.20804 | -1.29601 | 0.328928 |
| B1_ETA[30] | 0.037142 | 1.000064 | 10158.21 | 6198.866 | 189.5722 | -0.0991 | 0.17735 | -2.66808 | 0.209426 |
| B1_ETA[31] | 0.036214 | 1.000273 | 8503.157 | 5855.991 | 198.9915 | -0.108 | 0.174187 | -2.98212 | 0.207905 |
| B1_ETA[32] | -0.00773 | 1.000122 | 3983.003 | 5548.487 | -944.721 | -0.15166 | 0.130082 | 19.61105 | -0.05945 |
| B1_ETA[33] | -0.01478 | 1.000295 | 4133.341 | 5639.77 | -484.906 | -0.16488 | 0.117224 | 11.15559 | -0.12608 |
| B1_ETA[34] | 0.034544 | 1.000006 | 9002.611 | 6411.165 | 206.1925 | -0.10415 | 0.174235 | -3.01505 | 0.198262 |
| B1_ETA[35] | -0.08176 | 1.000215 | 7106.771 | 5981.548 | -88.0281 | -0.2228 | 0.058998 | 2.725195 | -1.38577 |
| B1_ETA[36] | 0.12507 | 1.000174 | 4250.428 | 6026.371 | 60.98489 | -0.03145 | 0.267015 | -0.25144 | 0.468401 |
| B1_ETA[37] | 0.176872 | 1.001066 | 2788.113 | 4592.223 | 48.78094 | -0.00271 | 0.330682 | -0.01535 | 0.534869 |
| B1_ETA[38] | 0.021998 | 1.000055 | 7114.297 | 6275.271 | 327.8354 | -0.12488 | 0.160739 | -5.67679 | 0.136856 |
| B1_ETA[39] | -0.01739 | 0.99989 | 10129.66 | 5971.301 | -398.086 | -0.15066 | 0.120494 | 8.662256 | -0.14434 |
| B1_ETA[40] | -0.00608 | 1.000586 | 6202.775 | 6158.961 | -1162.78 | -0.13893 | 0.133042 | 22.84616 | -0.04571 |
| B1_ETA[41] | 0.033009 | 0.999817 | 10174.62 | 5631.022 | 216.3259 | -0.10528 | 0.171939 | -3.1895 | 0.191982 |
| B1_ETA[42] | 0.120516 | 1.000109 | 8557.708 | 5882.579 | 61.91678 | -0.0288 | 0.263593 | -0.239 | 0.457206 |
| B1_ETA[43] | 0.072816 | 1.000182 | 8975.173 | 6156.948 | 99.52633 | -0.07549 | 0.205711 | -1.03674 | 0.353972 |
| B1_ETA[44] | -0.1208 | 1.000664 | 6934.944 | 5865.465 | -59.3442 | -0.26372 | 0.015738 | 2.183037 | -7.67595 |
| B1_ETA[45] | 0.081962 | 1.000342 | 4165.588 | 5350.863 | 91.63415 | -0.06544 | 0.225546 | -0.79848 | 0.363394 |
| B1_ETA[46] | -0.01919 | 1.000856 | 10046.19 | 6748.764 | -369.275 | -0.17088 | 0.107366 | 8.904327 | -0.17874 |
| B1_ETA[47] | -0.11396 | 0.999863 | 8683.262 | 5584.236 | -64.9822 | -0.26665 | 0.02305 | 2.339918 | -4.94396 |
| B1_ETA[48] | 0.19894 | 1.000843 | 3907.52 | 5200.437 | 40.88443 | 0.03183 | 0.346891 | 0.159999 | 0.573495 |
| B1_ETA[49] | 0.112769 | 1.000237 | 4586.516 | 5690.345 | 67.34634 | -0.04202 | 0.253772 | -0.37261 | 0.444373 |
| B1_ETA[50] | -0.08912 | 0.999994 | 9587.73 | 6287.372 | -82.7733 | -0.23537 | 0.054399 | 2.641173 | -1.63817 |
| B2_ETA[1] | -0.00016 | 1.001957 | 13511.15 | 6151.643 | -36816.2 | -0.11989 | 0.129359 | 752.2499 | -0.00123 |
| B2_ETA[2] | -0.03456 | 1.000278 | 2740.938 | 5088.492 | -199.377 | -0.2074 | 0.064102 | 6.001212 | -0.53912 |
| B2_ETA[3] | -0.01647 | 1.002602 | 4903.183 | 5267.004 | -384.503 | -0.16987 | 0.087146 | 10.31458 | -0.18898 |
| B2_ETA[4] | -0.00737 | 1.00094 | 8276.625 | 5168.71 | -825.352 | -0.14798 | 0.101193 | 20.07487 | -0.07284 |
| B2_ETA[5] | -0.02127 | 1.001018 | 5578.427 | 6085.382 | -300.568 | -0.16448 | 0.095342 | 7.733919 | -0.22307 |
| B2_ETA[6] | 0.009503 | 1.000646 | 6745.073 | 6332.612 | 645.977 | -0.10201 | 0.148236 | -10.7346 | 0.064106 |
| B2_ETA[7] | -0.0174 | 1.001703 | 5363.766 | 5232.008 | -360.494 | -0.16403 | 0.092578 | 9.427241 | -0.18794 |
| B2_ETA[8] | -0.03123 | 1.000674 | 2389.93 | 4403.061 | -228.144 | -0.20875 | 0.07046 | 6.683215 | -0.4433 |
| B2_ETA[9] | 0.00503 | 1.001404 | 7001.836 | 4478.408 | 1218.384 | -0.10966 | 0.143059 | -21.803 | 0.035158 |
| B2_ETA[10] | 0.022707 | 1.001625 | 3880.853 | 5044.911 | 288.1689 | -0.07878 | 0.186754 | -3.46928 | 0.121586 |
| B2_ETA[11] | -0.0095 | 1.001211 | 6957.627 | 6010.157 | -643.904 | -0.1511 | 0.101307 | 15.91165 | -0.09374 |
| B2_ETA[12] | -0.01176 | 1.000735 | 9392.897 | 5652.718 | -526.558 | -0.15527 | 0.099629 | 13.19819 | -0.11808 |
| B2_ETA[13] | 0.010266 | 1.000859 | 8135.715 | 6392.22 | 599.5548 | -0.10734 | 0.145925 | -10.4558 | 0.070352 |
| B2_ETA[14] | -0.00775 | 1.000368 | 11489.1 | 5737.22 | -804.777 | -0.14987 | 0.112243 | 19.34906 | -0.06901 |
| B2_ETA[15] | -0.02915 | 1.000953 | 4490.275 | 5065.67 | -229.42 | -0.17575 | 0.093602 | 6.029316 | -0.31142 |
| B2_ETA[16] | 0.017788 | 1.001268 | 2160.672 | 2521.595 | 376.0711 | -0.08497 | 0.184253 | -4.77672 | 0.09654 |
| B2_ETA[17] | -0.00695 | 1.0004 | 11651.5 | 5900.393 | -853.196 | -0.13742 | 0.106886 | 19.78163 | -0.06499 |
| B2_ETA[18] | 0.037071 | 1.001195 | 3081.422 | 5805.482 | 178.2114 | -0.05903 | 0.198054 | -1.59239 | 0.187177 |
| B2_ETA[19] | 0.028546 | 1.000853 | 1928.532 | 2772.483 | 249.631 | -0.0856 | 0.194986 | -2.9986 | 0.1464 |
| B2_ETA[20] | -0.0088 | 1.001409 | 10364.62 | 6271.956 | -705.705 | -0.1497 | 0.108786 | 17.00332 | -0.08093 |
| B2_ETA[21] | -0.02062 | 1.00051 | 4117.355 | 5305.029 | -305.773 | -0.16613 | 0.090478 | 8.055937 | -0.22792 |
| B2_ETA[22] | -0.02051 | 1.001403 | 5073.891 | 4525.071 | -314.138 | -0.1726 | 0.089652 | 8.416408 | -0.22875 |
| B2_ETA[23] | -0.04035 | 1.001385 | 2565.851 | 4974.26 | -179.604 | -0.21067 | 0.068676 | 5.220981 | -0.58755 |
| B2_ETA[24] | 0.048423 | 1.000702 | 2439.965 | 4820.169 | 147.193 | -0.04907 | 0.220911 | -1.01326 | 0.219199 |
| B2_ETA[25] | 0.005315 | 1.00125 | 11328.19 | 6086.728 | 1137.821 | -0.11034 | 0.139153 | -20.7604 | 0.038196 |
| B2_ETA[26] | 0.010772 | 1.000311 | 8279.757 | 5293.371 | 569.2477 | -0.09799 | 0.152247 | -9.09613 | 0.070756 |
| B2_ETA[27] | 0.001659 | 1.000453 | 13981.33 | 6118.716 | 3629.625 | -0.12821 | 0.123942 | -77.2981 | 0.013382 |
| B2_ETA[28] | 0.003291 | 1.000966 | 10384.95 | 6077.892 | 1803.01 | -0.11092 | 0.135299 | -33.7046 | 0.024324 |
| B2_ETA[29] | -0.04081 | 1.000517 | 2176.215 | 3785.174 | -181.267 | -0.21315 | 0.071771 | 5.223375 | -0.56858 |
| B2_ETA[30] | 0.023484 | 1.000547 | 4307.609 | 6520.151 | 267.807 | -0.07946 | 0.173358 | -3.38347 | 0.135468 |
| B2_ETA[31] | -0.01037 | 1.001453 | 8678.767 | 5544.071 | -589.21 | -0.14773 | 0.105725 | 14.24341 | -0.0981 |
| B2_ETA[32] | 0.048726 | 1.001548 | 1754.595 | 3908.713 | 152.6761 | -0.05078 | 0.226636 | -1.04212 | 0.214999 |
| B2_ETA[33] | 0.064576 | 1.001474 | 1464.224 | 3950.138 | 126.9516 | -0.04472 | 0.249517 | -0.69246 | 0.258804 |
| B2_ETA[34] | -0.00662 | 1.002197 | 11687.51 | 5368.953 | -902.916 | -0.13566 | 0.112115 | 20.4767 | -0.05909 |
| B2_ETA[35] | -0.00357 | 1.000523 | 12333.48 | 6189.3 | -1657.75 | -0.12706 | 0.123526 | 35.57664 | -0.02891 |
| B2_ETA[36] | -0.00643 | 1.000863 | 7403.11 | 5645.915 | -938.795 | -0.14513 | 0.106181 | 22.5806 | -0.06053 |
| B2_ETA[37] | -0.05026 | 1.002113 | 1599.734 | 4195.503 | -154.741 | -0.22688 | 0.06584 | 4.513733 | -0.76343 |
| B2_ETA[38] | 0.042378 | 1.000832 | 2225.794 | 4061.296 | 163.4766 | -0.05866 | 0.206481 | -1.38429 | 0.205238 |
| B2_ETA[39] | 0.001506 | 0.999969 | 12085.85 | 6429.954 | 3896.324 | -0.11408 | 0.129005 | -75.7301 | 0.011677 |
| B2_ETA[40] | 0.024354 | 1.001536 | 3181.663 | 4550.069 | 266.5065 | -0.07439 | 0.183773 | -3.05444 | 0.132523 |
| B2_ETA[41] | 0.012217 | 1.000456 | 10553.29 | 5787.365 | 483.4419 | -0.08824 | 0.147612 | -7.2223 | 0.082765 |
| B2_ETA[42] | 0.045248 | 1.000872 | 2639.936 | 5815.239 | 155.4895 | -0.05892 | 0.204714 | -1.30221 | 0.221031 |
| B2_ETA[43] | 0.007779 | 1.000266 | 11783.85 | 5766.828 | 762.7895 | -0.10159 | 0.143528 | -13.0599 | 0.054196 |
| B2_ETA[44] | -0.00154 | 1.000929 | 12702.57 | 5971.337 | -4000.76 | -0.13494 | 0.122091 | 87.90146 | -0.01257 |
| B2_ETA[45] | -0.01966 | 1.001011 | 3241.045 | 4737.674 | -334.316 | -0.18139 | 0.084417 | 9.225936 | -0.23291 |
| B2_ETA[46] | -0.02802 | 1.001603 | 3640.861 | 5393.813 | -241.785 | -0.19452 | 0.07241 | 6.942119 | -0.38696 |
| B2_ETA[47] | -0.0436 | 1.001256 | 2273.877 | 4934.177 | -165.777 | -0.2179 | 0.06151 | 4.997457 | -0.70885 |
| B2_ETA[48] | 0.017599 | 1.000423 | 8710.896 | 6242.051 | 344.8718 | -0.0926 | 0.155264 | -5.26139 | 0.113352 |
| B2_ETA[49] | -0.01532 | 1.000532 | 4768.741 | 4980.697 | -415.003 | -0.16575 | 0.093596 | 10.81892 | -0.16368 |
| B2_ETA[50] | -0.02183 | 1.000477 | 6373.059 | 6039.549 | -291.235 | -0.17511 | 0.081948 | 8.020675 | -0.26642 |

Table S4: individual parameters and random effects estimate for reparameterised SOE model

It is important to note that as the random effects are negative, without using the absolute value the RSE is also negative and this also impacts the 95CDIRAs values given that the 95%CDIs also include negative and positive values. However high precision would still fall under the values between either 0.7 and 1 or -0.7 and -1.
